# Impact of High Energy Milling and Mineral Additives on a Carbonate–Quartz–Apatite System for Ecological Applications

**DOI:** 10.3390/ma18153508

**Published:** 2025-07-26

**Authors:** Vilma Petkova, Katerina Mihaylova, Ekaterina Serafimova, Rositsa Titorenkova, Liliya Tsvetanova, Andres Trikkel

**Affiliations:** 1 Institute of Mineralogy and Crystallography “Acad. Ivan Kostov”, Bulgarian Academy of Sciences, Acad. G. Bonchev Str., bl. 107, 1113 Sofia, Bulgaria; 2 Department of Engineering Ecology, University of Chemical Technology and Metallurgy, 8 Kl. Ohridski Blvd., 1756 Sofia, Bulgaria; 3 Department of Materials and Environmental Technology, Tallinn University of Technology, Ehitajate tee 5, 19086 Tallinn, Estonia

**Keywords:** high energy milling activation, chemical reactivity, carbonate apatite, isomorphic substitution

## Abstract

In this study, high-energy milled (HEM) samples of natural phosphorites from Estonian deposits were investigated. The activation was performed via planetary mill with Cr-Ni grinders with a diameter of 20 mm. This method is an ecological alternative, since it eliminates the disadvantages of conventional acid methods, namely the release of gaseous and solid technogenic products. The aim of the study is to determine the changes in the structure to follow the solid-state transitions and the isomorphic substitutions in the anionic sub-lattice in the structure of the main mineral apatite in the samples from Estonia, under the influence of HEM activation. It is also interesting to investigate the influence of HEM on structural-phase transformations on the structure of impurity minerals-free calcite/dolomite, pyrite, quartz, as well as to assess their influence on the thermal behavior of the main mineral apatite. The effect of HEM is monitored by using a complex of analytical methods, such as chemical analysis, powder X-ray diffraction (PXRD), wavelength-dispersive X-ray fluorescence (WD-XRF) analysis, and Fourier-transformed infrared (FTIR) analysis. The obtained results prove the correlation in the behavior of the studied samples with regard to their quartz content and bonded or non-bonded carbonate ions. After HEM activation of the raw samples, the following is established: (i) anionic isomorphism with formation of A and A-B type carbonate-apatites and hydroxyl-fluorapatite; (ii) solid-phase synthesis of calcium orthophosphate-CaHPO_4_ (monetite) and dicalcium diphosphate-β-Ca_2_P_2_O_7_; (iii) enhanced chemical reactivity by approximately three times by increasing the solubility via HEM activation. The dry milling method used is a suitable approach for solving technological projects to improve the composition and structure of soils, increasing soil fertility by introducing soluble forms of calcium phosphates. It provides a variety of application purposes depending on the composition, impurities, and processing as a soil improver, natural mineral fertilizer, or activator.

## 1. Introduction

Phosphate rocks are a valuable resource for the fertilizer production industry, as they are the main source of phosphorus, whose quantity is limited on a global level. Around 90% of mined phosphorus is used for the manufacture of fertilizer [1].

**Figure 1:**
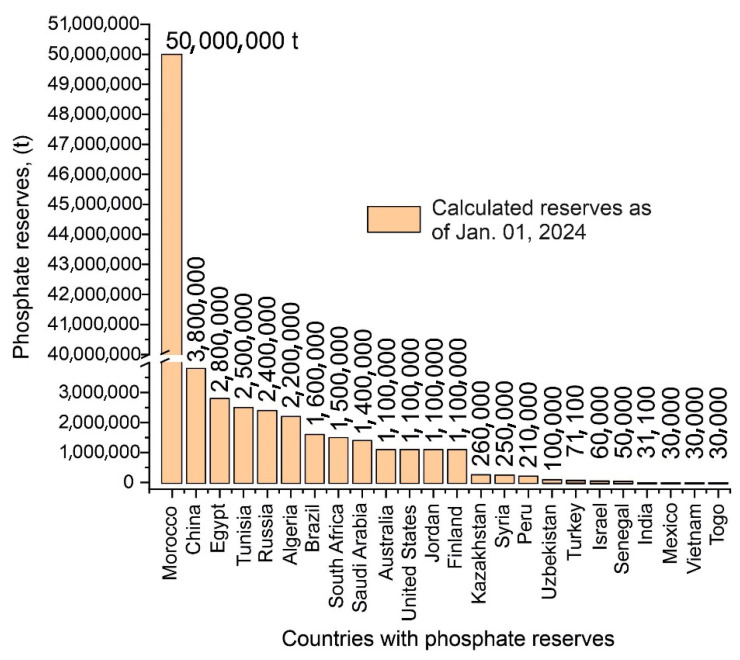
World phosphate rock reserves data for 2024 based on data from [2].

The world reserves of phosphate rocks are mainly distributed in the North African countries of Morocco, Tunisia, Algeria, and Egypt. Morocco is the country with the largest reserves, equivalent to 50 trillion tons. Phosphate resources of economic importance are also found in the USA, Russia, China, and Australia. Their quantities are many times smaller than the reserves of North African countries, as illustrated in Figure 1.

In addition to phosphorus, phosphate rocks contain rare earth elements (REEs) that are crucial for the current energy transition and are regarded as having the highest supply risk for the European Union (EU) [3]. Since 2020, their importance has become even greater when the European Commission recognized them along with phosphorus and REEs as critical raw materials (CRMs) [4].

**Figure 2:**
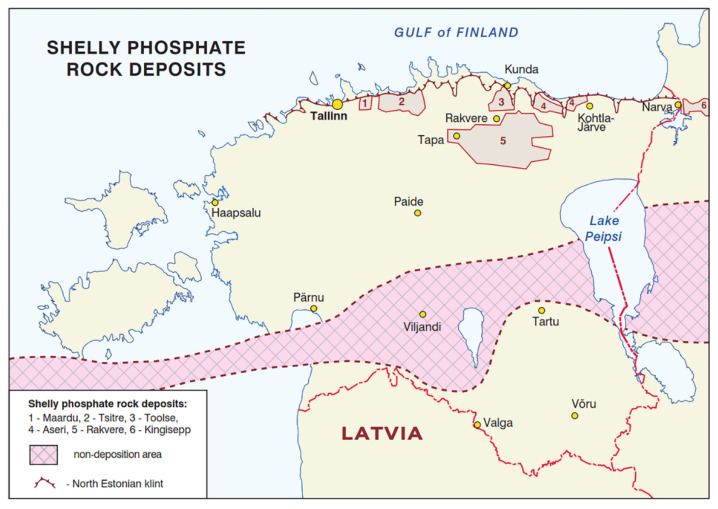
Phosphorite ore deposits in Estonia [6].

The largest phosphate rock deposits found within the EU’s borders are Estonian phosphorites. Their total reserves are estimated to be approximately 3 billion metric tons, out of which 820 million metric tons are P_2_O_5_ (Figure 2) [1,5,6].

The phosphorite’s P_2_O_5_ content in the different deposits changes between 6% and 20%, along with the type and amount of impurity minerals [1]. The phosphorite itself varies in grade from low- to medium and high-grade, where the mineralization is carried by the apatite group minerals-Ca_5_(PO_4_)_3_(OH,F,Cl). The most common apatite found in the Estonian phosphorite ores is carbonate fluorapatite (CAF-apatite), also known as francolite. Francolite is a non-stoichiometric mineral that varies in its structure and chemistry, owing to the possible chemical substitutions that can occur in all its lattice sites—the two Ca^2+^ sites, F^−^ and PO_4_^3−^ [7].

The industrial importance of phosphate raw materials is relevant for the production of the nowadays important phosphate fertilizers and mineral acids. The main approach to the treatment of phosphate materials is acid-based due to the chemical stability of the base mineral in them, apatite [8,9,10]. Their processing is further complicated by the presence of impurity minerals, such as calcite (CaCO_3_), dolomite (CaMg(CO_3_)_2_) illite K_0.6–0.85_(Al,Mg)_2_(Si,Al)_4_O_10_(OH)_2_, muscovite (KAl_2_(AlSi_3_O_10_)(OH)_2_), chamosite (Fe^2+^,Mg,Al,Fe^3+^)_6_(Si,Al)_4_O_10_(OH,O)_8_), K-feldspar (KAlSi_3_O_8_), pyrite (FeS_2_), as well as rare earth and critical elements [11]. The application of classical acid methods is associated with the processing of high-quality phosphate raw materials with a content of the main component in the form of P_2_O_5_-25–30%. Their main disadvantage is the production of large volumes of solid-phase waste with the main component CaSO_4_, known by the name of phosphogypsum due to the presence of an impurity of P_2_O_5_ [12]. Another equally significant disadvantage of acid processing is the pollution of the atmosphere with acid and greenhouse gases. Solid-phase waste also removes essential biogenic nutrients such as Ca, S, and P from the nutrient cycle, and the gaseous emissions contribute to increasing the ecological footprint of these industries and deteriorating air quality, which is increasingly unacceptable in view of compliance with the principles of a circular economy, environmental legislation, and depleting natural resources. All of the above problems also correlate with the real economic situation in the world of high consumption, the use of natural energy sources, and increasing demand for food and energy. This means that environmental constraints and circular economy intersect with the growing needs of feeding populations in the face of scarcity of raw materials and energy sources. The production of fertilizers is therefore still relevant in response to growing food needs, but this is also linked to reducing the ecological footprint of this highly polluting production. Innovations in modern research are linked on one hand to the application of environmentally friendly technologies and on the other, to the processing of lower quality raw materials, the use of activators, waste, and alternative sources of raw materials and energy for the production of fertilizers and soil improvers.

In the field of methods and technologies, various authors have investigated the mechanochemical treatment of phosphorites, focusing on different aspects: activation conditions (variation in the activation time, size and material of the grinding bodies) and P_2_O_5_ solubility [13,14,15,16,17,18,19,20]; structural and chemical changes occurring in the phosphorite after activation [21,22,23,24,25].

This study is a continuation of the team’s previous work studying the composition and properties of samples from Estonian phosphorite ore deposits [5,25]. Previous research conducted [5,25] aimed to characterize the composition of phosphorite ore from various deposits in Estonia-Iru, Toolse, Ülgase, and Rakvere deposits by identifying the main minerals in the ore-carbonate-rich fluorapatite (francolite) and quartz, as well as the impurity minerals-calcite, dolomite, muscovite, illite, chamosite, sanidine, orthoclase, and pyrite. Microstructural studies were carried out to trace the composition and impurity minerals in different ground layers of the ore, phase composition, isomorphic substitutions, and thermal behavior.

This study investigates the possibility of processing samples from the studied deposits by acid-free methods. In the area of raw materials and modification of their properties, alternative approaches are being sought to enhance chemical activity and reactivity with appropriate enhancers and activators, as well as impact on structure-phase transformations, solid-phase reactions and all appropriate approaches in the search for resource recovery systems, introducing effective, efficient and socially acceptable measures and practices to integrate environmental and climate change policies.

The aim of the study is to determine the choice of method and the appropriate conditions for phosphorite ore treatment under which the reactivity of the main mineral apatite and mineral additives on the carbonate–quartz–apatite system for ecological applications is increased, acid-free preparation of improvers and fertilizers, raw material for REE extraction, soil improvers, activators, etc. Increase in reactivity is determined by structural-phase transformations in the structure of apatite and impurity minerals, which justifies determining the changes in the structure to follow the solid-state transitions and the isomorphic substitutions in the anionic sub-lattice in the structure of the main mineral apatite in the samples from Estonia, under the influence of HEM activation. It is also interesting to investigate the influence of HEM on structural-phase transformations on the structure of impurity minerals-free calcite/dolomite, pyrite, and quartz.

## 2. Materials

In the present work, samples from the Toolse and Kabala deposits in Estonia are studied.

Some of the largest and most important phosphorite deposits in Estonia are Toolse and Rakvere [5,7]. In Toolse, the phosphate ore body encompasses quartzose phosphatic shelly sandstones whose thickness, together with the phosphatic shelly brachiopods, ranges from 1.5 to 7.9 m. The average P_2_O_5_ content for the producing bed is 10.6%, usually varying between 4 and 28%. The average silica content is 70.9%, ranging between 21.6% and 88.2%, while the average Fe_2_O_3_ is 1.5%, ranging between 0.2 and 5.0%. The deposit is divided into 4 ore types based on their physical and chemical characteristics: (1) shelly phosphorite ore, (2) shelly phosphorite ore with high quartz sand content, (3) weathered shelly phosphorite ore, and (4) shelly phosphorite with an elevated Fe content. The estimated P_2_O_5_ reserves are around 27 million metric tons [6].

With its estimated 700 million tons of P_2_O_5_, the Rakvere shelly phosphorite deposit is regarded as the largest one in Europe. The thickness of the phosphate ore ranges between 3.1 and 7.6 m. while the ore’s P_2_O_5_ values are between 7.1 and 14.9%. The highest values were found in the eastern part of the Rakvere deposit, at the prospective Kabala mining field. The ore from the deposit is relatively pure-undesirable elements such as Fe and Mg are low in content: Fe_2_O_3_ is present in amounts up to 8.75%, while MgO averages at 1.21% [6]. In the Kabala deposit, Fe occurs as part of the mineral goethite (F^3+^O(OH)), while in Toolse it is present in the form of pyrite (FeS_2_) [7].

Overall, Estonian phosphorites are well-suited for use in the production of fertilizers as they contain low amounts of Cd (1–5 ppm) and U (~50 ppm). Both elements are typically present in phosphate rocks and, due to their toxicity, need to be removed before the production process [26].

In the present study, the mechanochemical activation method was used to improve the chemical reactivity of phosphorite ores from two deposits in Estonia. The method is used to modify the properties of substances with lower reactivity in order to intensify them; obtain nanosized materials with new properties; solid-phase syntheses, etc. Such treatment of the substance is called mechanoactivation, or triboactivation (in dry mode), and is carried out by so-called energy-transferring devices, such as planetary, vibrating, jet mills, disintegrators, etc., where high frequencies and the force of the mechanical impact are combined. Its application leads to the accumulation of structural defects, distortion, and an increase in specific and geometric grain surface area, solid-phase synthesis, phase transitions, and amorphization. When the process is accompanied by chemical reactions, the method is known by the name of mechanochemistry or tribochemistry. A variation of the term “triboactivation” is the definition “high energy mill”, which will also be used in this publication. The effects on the treated material at the submicron scale are the subject of study of microstructural analysis and methods for this include electron microscopy, powder X-ray diffraction, infrared spectroscopy, thermal methods, etc., the results of which will also be used in this publication.

The initial sample of the Toolse phosphorite is named To0, and that of the Kabala phosphorite-Ka0. The activated samples with milling times: 10, 30, 60, 120, and 240 min were named To10, To30, To60, To120, and To240 and Ka10, Ka30, Ka60, Ka120, and Ka240, respectively.

## 3. Methods

HEM activation was carried out in a planetary mill Pulverisette-5, Fritsch Company (Idar-Oberstein, Germany), activation time from 10 min to 240 min, sample mass: 20 g. Triboactivation was carried out at 320 rpm to ensure maximum treatment effect. For the same reason, high-density Cr-Ni stainless steel grinding balls with a large diameter of 20 mm and grinders of 250 mL volume were used. The weight of the grinding balls is 510 g. The ratio between the grinding bodies and the samples’ mass is 25.5:1 at 50% of the grinders’ volumes. Treatment was conducted in an air atmosphere.

In order to evaluate the transition degree of the unassimilated forms of P_2_O_5_ to assimilated (P_2_O_5_^a^) as a result of the HEM activation, a standard chemical method has been used for characterizing the dissolution of the phosphorus fertilizers in 2% citric acid. The standardized methods for the determination of P_2_O_5_^a^ are the following:

− Bulgarian National Standard 14131-1988, according to which P_2_O_5_^a^ can be determined by direct extraction with 2% citric acid [27];
− Instruction from Directive 77/535/EEC, p. 3.1.4 “Extraction of phosphorus soluble in neutral ammonium citrate” where the extraction of P_2_O_5_^a^ is performed by direct extraction [28].

The specific surface area (SSA) measurements were performed by the Brunauer–Emmett–Teller (BET) method (adsorptive gas N_2_, carrier gas He, heating temperature 150 °C) using a sorptometer EMS-53 (EAK, Tallinn, Estonia) and KELVIN 1040/1042 software v. 3.12 (Costech International, Pioltello, Italy).

The experimental data for SSA and P_2_O_5_^a^/P_2_O_5_^tot^ for all samples were mathematically processed in order to achieve the best R fit (as close to 1 as possible) with a given function.

Wavelength Dispersive X-ray fluorescence (WD-XRF) analysis was performed using a spectrometer WD-XRF Super-mini 200—Rigaku (Osaka, Japan), operating at 50 kV and 4 mA, a 200 W X-ray tube with a Pd-anode, 30 mm^2^, and a vacuum atmosphere. Two different X-ray detectors were used: a gas flow proportional counter for light elements (Detector 1, P-10 gas) and a scintillation counter for heavy elements (Detector 2, Nal crystal). Three analyzing crystals were used (according to the wavelength range): LIF 200 (for Ti-U), PET (for Al-Ti), and RX25 (for F-Mg). The samples were prepared as tablets with CERE-OX-BM-0002-1 powder (Environmental XPRT, Madrid, Spain). The sample and binder were combined in a mass ratio of 5:1 (5 g sample to 1 g binder). Rigaku’s built-in software package “ZSX” v.7.67 was used for the processing of the data.

The powder X-ray diffraction (PXRD) measurements were made by Empyrean Powder X-ray diffractometer (Malvern Panalytical, Almelo, The Netherlands) (with multi-channel detector Pixel 3D), CuKα radiation (λ = 0.15418 nm) (operating at 45 kV, 40 mA) from 5 to 70° 2θ with a step of 0.026° (ground sample with weight—1.0 ± 0.1 mg). The PDF database [29] was used for the determination of the phases and minerals present in the samples. The free and open-source software package Profex v.5.5.0/17.05.2025 was used for pattern refinement of the crystal structure [30,31].

The Fourier Transform Infra-Red (FTIR) spectra were registered on a Bruker Tensor 37 (Bruker, Berlin, Germany) spectrometer in the range 400–4000 cm^−1^. Infrared spectra were collected on transparent pellets prepared using potassium bromide (KBr). The weighted sample in the range 1–2 mg was mixed with 200–300 mg of KBr (spectroscopic grade), previously dried at 120 °C. A resolution of 2 cm^−1^ was used, collecting 60 scans for each sample.

## 4. Results

### 4.1. Wavelength-Dispersive XRF Analysis

Studies were conducted on the contents of the major chemical elements that characterize the composition of a series of inactivated and triboactivated (0–240 min) samples from the Toolse and Kabala deposits by wavelength-dispersive X-ray fluorescence analysis. The results are presented in Table 1 for the Toolse series and in Table 2 for the analogous samples from the Kabala deposit.

Table 1 also presents the XRF measurements of the major components of the activated samples’ composition.

The XRF analysis results of the major chemical element contents of the Toolse phosphorite confirm the studies presented in previous publications but complement the data with results for other chemical elements and their concentrations [20]. At the same time, data on the content of the main elements in the triboactivated samples were obtained, which makes it possible to trace the change dynamics under the influence of the applied triboactivator planetary mill treatment.

The XRF results present samples from both deposits with a suitable composition for their industrial application, for example, for the production of phosphorus fertilizers, taking into account that the phosphorus content in the form of P_2_O_5_^tot^ varies in the range of 25–29%. For the P content as total P_2_O_5_, the data from the analyses revealed a slight decrease of 5.38% in the P content for Toolse (from 26.77% to 25.33%). For Kabala, an increase to 29% was found at 10–30 min as a result of high-energy mechanochemical treatment, after which a slight decrease was recorded.

The CaO content remains stable with minimal fluctuations in both sample series (~49% for Toolse and ~54% for Kabala). A slight decrease is found in some of the samples, with Ca^2+^ being stable in the apatite structure. Most likely, some of it is involved in isomorphous substitutions, but no significant change in mass content.

The presence of quartz above 10% in the compositions of both sets of samples is estimated to be high. SiO_2_ shows moderate variations, with a slight increase found in the Kabala samples. The SiO_2_ and Fe_2_O_3_ contents are attributed to the presence of minerals such as quartz and pyrite [5]. Fe_2_O_3_ has minor variations, probably caused by amorphization of pyrite due to HEM treatment. The observed fluctuations are probably the result of incomplete homogenization, local quartz destruction, or agglomeration.

**Figure 3:**
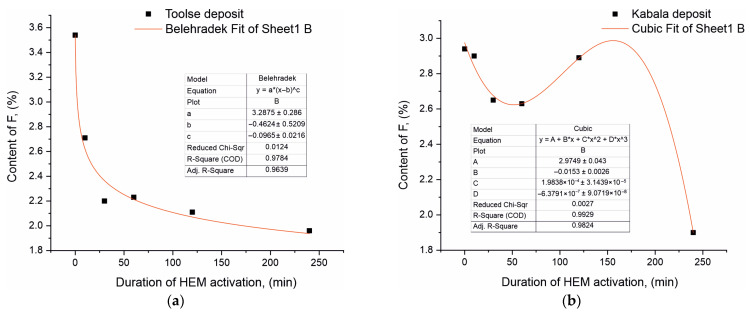
(**a**) F content dependence in Toolse phosphorite ore on the triboactivation duration in the time 0–240 min. (**b**) F content dependence in Kabala phosphorite ore on the triboactivation duration in the time 0–240 min.

The most significant changes were in the light element F content, which for both sample series decreased with increasing treatment time in the triboreactor. Thus, for the Toolse series, the F content in the non-activated To0 sample was 3.54% and decreased to 1.96% at To240, or by 44.63%. In the activated Kabala samples, the corresponding decrease in F content was 35.37% from 2.94% at To0 to 1.90% at To240. In contrast to the contents of Ca, P, and Si who are relatively constant with slight fluctuations, the content decrease keeps a stable trend in the case of F. To illustrate the results even more clearly, a linearization of the data obtained from the XRF studies for the F content was carried out and presented in Figure 3a,b.

The experimental data regarding the F content in both sample series was mathematically processed in order to achieve the best R fit (as close to 1 as possible) with a given function. The mathematical processing of the experimental results for the F-concentration versus triboactivation duration for the two series of samples is of a nonlinear nature. The results for the Toolse series are best described by a step function of the form:

(1) $$y=a*{\left(x-b\right)}^{c},$$

where *y* = 3.2875 ∗ (*x* + 0.4624)^0.0965^. The coefficient “a” is a constant multiplier and has the meaning of the initial value of the function *y*, and when *x* = 0, then *y* = a ∗ (0 − b)^c^ = a ∗ (−b)^c^. When parameter “b” and parameter “c” are close to 1, then function “(−b) ∗ c” is close to 1 and function *y* ≈ a, which means the numerical value of the F content in the initial sample before triboactivation.

As the activation time increases, the significance of the coefficients “b” and “c” have an offset from the initial value—b and a power parameter—c. Since c = 0.0965 is a positive value between 0 and 1 (0 < c < 1), this indicates a decreasing rate of increase and in combination with the offset (*x* + 0.4624) has a decreasing function. The dependence is down-sloped and has a decreasing significance which is consistent with the experimental data from the XRF analysis for the Toolse series.

The results for the Kabala samples are described by a complex polynomial function for nonlinear dependence between *x* and *y* of the form:

(2) $$y=a+b*x+c{\;*\;x}^{2\;}+\;d*x^{3},$$

where *y* = 2.9749 − 0.0153 ∗ *x* + 1.9838 ∗ 10^−4^ ∗ *x*^2^ − 6.3791 ∗ 10^−7^ ∗ *x*^3^. It is characterized by a free term, a, which gives the significance of the maximum value of the function *y* at *x* = 0 and gradually decreases with increasing values of *x*—triboactivation duration, i.e., it has a decreasing significance, thus confirming the experimental results of the XRF analysis of the F content for the Kabala series.

MgO, SO_3_, Na_2_O, K_2_O: Almost constant values are observed for MgO. For SO_3_, a slight increase is observed, which is possible upon release from pyrite or formation of new sulfate phases. Fluctuations in Na_2_O content were not linear, but a slight increasing trend was observed after triboactivation, especially for samples Ka10, Ka30 and Ka120. The increased content after activation is probably related to disaggregation and better detection of Na-bearing phases (e.g., Na-bearing clays or silicates). K_2_O shows a decreasing trend in all samples after activation, more pronounced in the Kabala samples (from 0.16 to 0.13%). This is probably related to degradation or transformation of K-bearing clays, which amorphize upon mechanochemical treatment. Potassium is separated from the crystal structure and becomes volatile. Since K^+^ is rarely involved in isomorphous replacement in apatite, therefore, a more likely cause is transformation or loss by mineral impurities [32].

### 4.2. Chemical Analysis for the Determination of Assimilable P_2_O_5_

**Figure 4:**
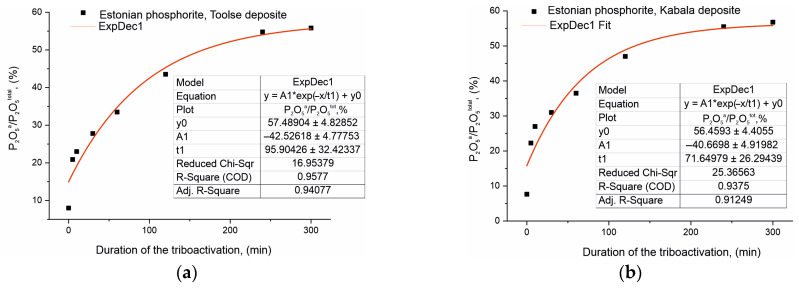
(**a**) Results of determination of soluble P_2_O_5_ forms in Toolse phosphorite in 2% citric acid. (**b**) Results of determination of soluble P_2_O_5_ forms in Kabala phosphorite in 2% citric acid.

Studies were carried out to determine the chemical activity of the activated Toolse and Kabala phosphorites to determine the soluble forms of P_2_O_5_—total and assimilable in phosphates. The standardized methods for the determination of P_2_O_5_^a^ are described in Section 3 and are based on direct extraction where the P_2_O_5_^a^ content is carried out in a 2% citric acid solution. The dependence of P_2_O_5_^a^/P_2_O_5_^tot^ (%) as a function of the duration of triboactivation was investigated and the results obtained are presented graphically in Figure 4a,b. The P_2_O_5_^a^/P_2_O_5_^tot^ (%) ratio is approximately the same for the initial non-activated samples of both series, 8.00% for To0 and 7.56% for Ka0, despite the fact that the phosphorite from Kabala has a higher total P_2_O_5_ content.

The solubility results of the assimilable P_2_O_5_^a^ for the activated samples can be conventionally divided into two segments. In the first part, up to 60 min of triboactivation, the P_2_O_5_^a^/P_2_O_5_^tot^ ratio (%) increases rapidly by a factor of 4.2 (from 8.00% to 33.5%) for the samples from the Toolse deposit and by a factor of 4.8 for the samples from the Kabala deposit. In the second part of the dependencies in Figure 4, after 60 min the increase is at a slower rate, respectively: 1.7 times (from 33.50% for To60 to 55.83% for To240) for the samples from the Toolse deposit, and 1.5 times resp. for the samples from the Kabala deposit (from 36.50% for Ka60 to 55.49% for Ka240).

The mathematical processing of the experimental data for P_2_O_5_^a^/P_2_O_5_^tot^ supports the nonlinear increase in the chemical solubility of P_2_O_5_^a^. The results for both sets of samples are best described by an exponential function of the form:

(3) $$y=y_{0}+A1*e^{\left(-\frac{x}{t1}\right)}$$

where *y* = 57.48904 − 42.52618 ∗ exp(*x*/95.90426) for the Toolse samples and *y* = 56.4593 − 40.6698 ∗ exp(*x*/71.64979) for the Kabala samples.

The mathematical models represent the exponential equations with empirical values of the coefficients and the free term. This exponential function describes the solubility variation as a function of triboactivation duration for the Toolse and Kabala samples. The values of y0 represent the values of the theoretical maximum solubility that can be reached for very prolonged triboactivation periods, i.e., high values along the *x*-axis. As the *x*-axis values (triboactivation time) increase, the exp(*x*/95.90426) term takes on increasingly larger values, but as it is subtracted from *y*0, this means that the *y*-axis values decrease. This is supported by the experimental results obtained, which verify a strong retardation of the solubility increase with increasing activation duration. The preexponential multiplier can be defined as a coefficient related to the scale of the solubility change. At values of *x* = 0, the exponential multiplier becomes equal to the pre-exponential coefficient, i.e., for the Toolse series at *x* = 0, *y* = 57.48904 − 42.52618 = 14.96286, and for the Kabala series at *x* = 0, *y* = 56.4593 − 40.6698 = 15.7895. The values obtained represent the initial solubility values without activation, which are close in significance to the real data.

The t1 coefficient values are taken as the time constant of the tribochemical activation. The higher the value of this coefficient, the more the solubility of P_2_O_5_ is slowed down, which is consistent with the experimental results.

These results are important for evaluating the effect of tribochemical activation on the properties of phosphorite samples and can serve to optimize processes related to their processing and application.

### 4.3. BET Specific Surface

**Figure 5:**
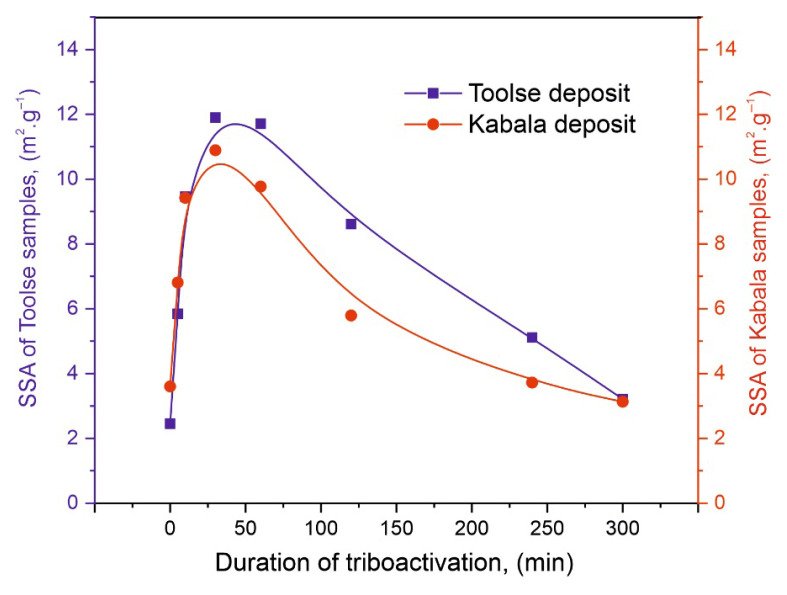
Results for specific surface area determination of non-activated and triboactivated Toolse and Kabala samples.

The specific surfaces of the same series of non-activated and activated samples were determined by the BET method. The results are presented graphically in Figure 5.

The measured values for the specific surface area (SSA) of the samples from the two localities are analogous to the P_2_O_5_ solubility results and are also analogous to each other in values and kinetics of variation. The initial SSA values for the Toolse deposit is 2.45 m^2^·g^−1^, while for the Kabala deposit, respectively 3.30 m^2^·g^−1^. Similar maximum SSA values were recorded at different triboactivation times for the two series, 11.71 m^2^·g^−1^ at 60 min for Toolse (To60) and 10.89 m^2^·g^−1^ at 30 min for Kabala (Ka30). SSA increases during 10, 30, and 60 min as the mechanical impact on the larger particles leads to the reduction of their size and the subsequent revealing of a fresh reactive surface. With increasing activation duration after 60 min, a strong decrease in values was recorded for both series, with SSA reaching 3.21 m^2^·g^−1^ and 3.13 m^2^·g^−1^ at 240 min, which almost matched the initial SSA values for the initial samples To0 and Ka0. The reduction of SSA after 60 min of activation is owed to particle agglomeration, which occurs due to the impact of electrostatic forces [20].

### 4.4. Powder X-Ray Phase Analysis

**Figure 6:**
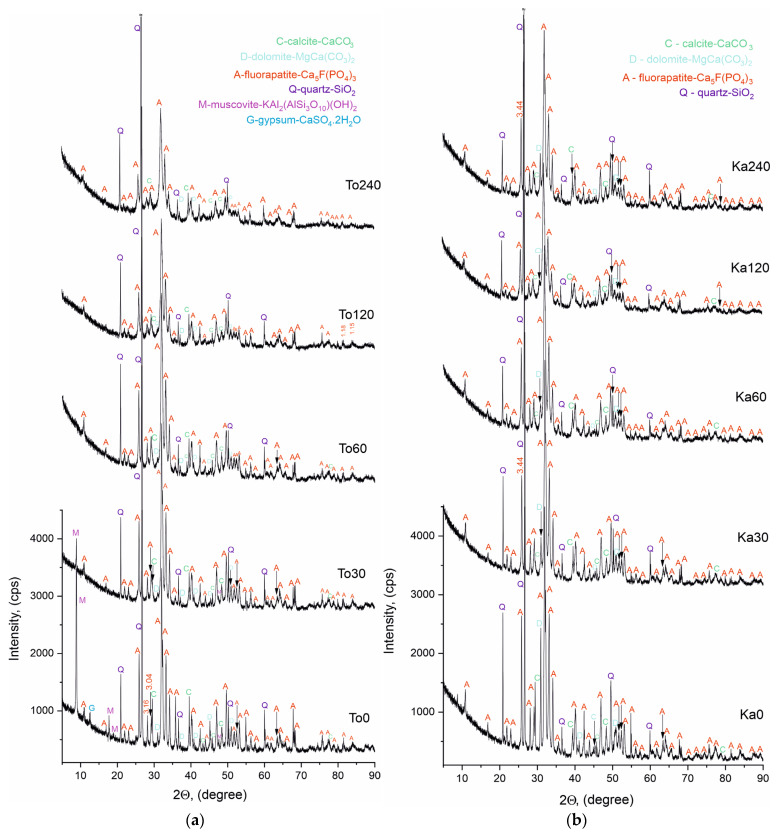
(**a**) XRD patterns of non-activated and triboactivated Toolse samples (30–240 min). (**b**) XRD patterns of non-activated and triboactivated Kabala samples (30–240 min).

Powder X-ray phase analysis (PXRD) was performed on both sets of samples to identify the phase composition before and after triboactivation. The results are presented in Figure 6a,b and Table 3, Table 4 and Table 5.

The Bragg reflections were identified in the diffractograms of the non-activated and triboactivated samples of the two phosphorite series from Toolse and Kabala, in accordance with the standards for each phase listed in Table 3. The identified phases matched for both series of samples are fluorapatite, calcite, dolomite and quartz. The common characteristic of the diffractograms is that they represent spectra of well-crystallized mineral phases with a high degree of crystallinity. The high and narrow peaks with high intensity and good background statistics are in evidence.

The phase composition of the triboactivated samples—To30 ÷ To240 and Ka30 ÷ Ka240 remains identical to the phases characterizing the non-activated initial compositions—To0 and Ka0. A difference is found with respect to the type of spectra, and it is related to a decrease in intensity and broadening of the base of the lines, better pronounced in the samples after triboactivation for 120 and 240 min. This effect is associated with a decrease in particle size resulting from grinding, leading to a decrease in the degree of crystallinity, which is the most likely reason for the absence of muscovite and gypsum lines in the diffractograms of the activated Toolse series samples. The observed pattern is well known and described in the literature in a number of studies by other authors, as well as in our previous publications [20,33,34].

**Figure 7:**
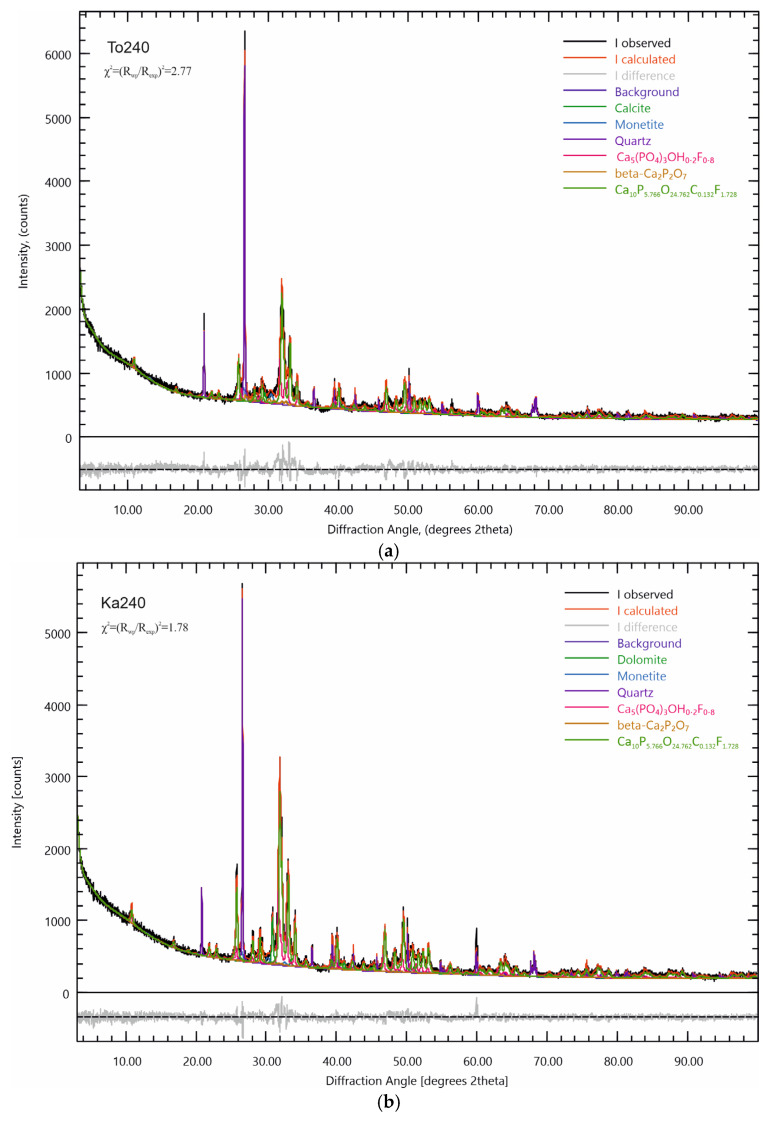
(**a**) PXRD pattern refinement of triboactivated for 240 min Toolse sample. (**b**) PXRD pattern refinement of triboactivated for 240 min Kabala sample.

At the base of the broadened diffraction lines, it is possible that low-intensity reflections of phases with close interplane distances overlap. For a more detailed analysis of the phase composition and refinement of the diffraction characteristics of the studied samples, the specialized open access software Profex v.5.5.0 was used [30,31]. Using this software package, Rietveld refinement was performed to refine the crystal-chemical parameters of the refined phases in the composition of the triboactivated samples. The results of the analysis are presented in Table 4 for the Toolse samples and Table 5 for the Kabala samples, and on Figure 7a,b. The Rietveld refinement of the To0-To120 and Ka0-Ka120 samples are given in the Supplementary Materials file. The numerical values for parameter χ2 (acceptance limit) range from 1.50 to 2.77.

**Table 4:** Refined chemical composition and structural parameters of the Toolse samples using Profex v.5.5.0 [30,31] with reference spectra data from crystallographic databases [35,36].

Samples	Identified Phase	Content	Lattice Parameters		
		%	a, nm	b, nm	c, nm
Reference	Ca_5_(PO_4_)_3_F Carbonate fluorapatite— Fluorapatite (code amcsd 0001256)	-	0.93973	0.93973	0.68782
To0	Ca_5_(PO_4_)_3_F Ca_9.7560_P_5.9520_O_23.9760_F_1.8840_	61.66	0.93588	0.93588	0.68923
To30	Ca_5_P_2.883_O_12.321_C_0.042_F_0.921_ (cod9010504) Ca_10_P_5.7660_O_24.6420_C_0.0840_F_1.8420_	49.49	0.93509	0.93509	0.68946
To60	Ca_5_P_2.883_O_12.381_C_0.066_F_0.864_ (cod9010506) Ca_10_P_5.7660_O_24.7620_C_0.1320_F_1.7280_	43.49	0.93440	0.93440	0.68895
To120	Ca_5_P_2.883_O_12.381_C_0.066_F_0.864_ (cod9010506) Ca_10_P_5.7660_O_24.7620_C_0.1320_F_1.7280_	39.86	0.93479	0.93479	0.68862
To240	Ca_5_P_2.883_O_12.381_C_0.066_F_0.864_ (cod9010506) Ca_10_P_5.7660_O_24.7620_C_0.1320_F_1.7280_	41.89	0.93550	0.93550	0.69058
Reference	Fluor hydroxyl apatite Ca_5_(PO_4_)_3_((OH)_0.2_F_0.8_) (cod1533064)	-	0.9374	0.9374	0.68826
To0	-	-	-	-	-
To30	Ca_5_(PO_4_)_3_((OH)_0.2_F_0.8_)	21.06	0.93925	0.93925	0.68949
To60	Ca_5_(PO_4_)_3_((OH)_0.2_F_0.8_)	26.83	0.93940	0.93940	0.69074
To120	Ca_5_(PO_4_)_3_((OH)_0.2_F_0.8_)	24.84	0.94040	0.94040	0.69144
To240	Ca_5_(PO_4_)_3_((OH)_0.2_F_0.8_)	17.13	0.94637	0.94637	0.68137
Reference	CaCO_3_-Calcite (code amcsd 0000098)	-	0.49900	0.49900	1.79172
To0	CaCO_3_	4.21	0.49844	0.49844	1.70510
To30	CaCO_3_	2.54	0.49630	0.49630	1.71220
To60	CaCO_3_	2.01	0.49660	0.49660	1.71240
To120	CaCO_3_	3.85	0.49710	0.49710	1.71000
To240	CaCO_3_	2.89	0.49500	0.49500	1.71000
Reference	CaMg(CO_3_)_2_-Dolomite (code amcsd 0000086)	-	0.48150	0.48150	1.61190
To0	CaMg(CO_3_)_2_	2.98	0.4799	0.4799	1.6100
To30	CaMg(CO_3_)_2_	2.27	0.4849	0.4849	1.5970
To60	-	-	-	-	-
To120	-	-	-	-	-
To240	-	-	-	-	-
Reference	SiO_2_-Quartz (code amcsd 0006362)	-	0.49137	0.49137	0.54047
To0	SiO_2_	22.53	0.49136	0.49136	0.54055
To30	SiO_2_	20.69	0.49151	0.49151	0.54069
To60	SiO_2_	19.03	0.49153	0.49153	0.54070
To120	SiO_2_	21.81	0.49148	0.49148	0.54061
To240	SiO_2_	21.91	0.49147	0.49147	0.54072
Reference	Al_11.68_Fe_0.32_K_2.40_Si_12_O_48_ Muscovite (code amcsd 0001080)	-	0.51890	0.90040	2.02560
To0	Al_11.68_Fe_0.32_K_2.40_Si_12_O_48_	8.63	0.5260	0.8754	2.0200
To30	Al_11.68_Fe_0.32_K_2.40_Si_12_O_48_	1.35	0.5236	0.8990	2.0198
To60	-	-	-	-	-
To120	-	-	-	-	-
To240	-	-	-	-	-
Reference	CaHPO_4_, Monetite	-	0.6910	0.6627	0.6998
To0	-	-	-	-	-
To30	CaHPO_4_	2.60	0.6881	0.6652	0.6970
To60	CaHPO_4_	3.73	0.6910	0.6586	0.6981
To120	CaHPO_4_	6.01	0.6940	0.6586	0.6981
To240	CaHPO_4_	8.75	0.6910	0.6586	0.6970
Reference	β-Ca_2_P_2_O_7_ (cod2001132)	-	0.66858	0.66858	2.41470
To0	-	-	-	-	-
To30	-	-	-	-	-
To60	β-Ca_2_P_2_O_7_	4.93	0.66189	0.66189	2.40500
To120	β-Ca_2_P_2_O_7_	3.62	0.66420	0.66420	2.40900
To240	β-Ca_2_P_2_O_7_	7.43	0.66580	0.66580	2.43885

**Table 5:** Refined chemical composition and structural parameters of the Kabala samples using Profex v.5.5.0 [30,31] with reference spectra data from crystallographic databases [35,36].

Samples	Identified Phase	Content	Lattice Parameters		
		%	a, nm	b, nm	c, nm
Reference	Ca_5_(PO_4_)_3_F Carbonate fluorapatite— Fluorapatite (code amcsd 0001256)	-	0.93973	0.93973	0.68782
Ka0	Ca_5_(PO_4_)_3_F Ca_9.7560_P_5.9520_O_23.9760_F_1.8840_	76.54	0.93627	0.93627	0.68926
Ka30	Ca_5_P_2.883_O_12.321_C_0.042_F_0.921_ (cod9010504) Ca_10_P_5.7660_O_24.6420_C_0.0840_F_1.8420_	52.11	0.93486	0.93486	0.68924
Ka60	Ca_5_P_2.883_O_12.381_C_0.066_F_0.864_ (cod9010506) Ca_10_P_5.7660_O_24.7620_C_0.1320_F_1.7280_	48.47	0.93484	0.93484	0.68891
Ka120	Ca_5_P_2.883_O_12.381_C_0.066_F_0.864_ (cod9010506) Ca_10_P_5.7660_O_24.7620_C_0.1320_F_1.7280_	39.41	0.93491	0.93491	0.68759
Ka240	Ca_5_P_2.883_O_12.381_C_0.066_F_0.864_ (cod9010506) Ca_10_P_5.7660_O_24.7620_C_0.1320_F_1.7280_	51.05	0.93514	0.93514	0.68914
Reference	Fluor hydroxyl apatite Ca_5_(PO_4_)_3_((OH)_0.2_F_0.8_) (cod1533064)	-	0.93740	0.93740	0.68826
Ka0	-	-	-	-	-
Ka30	Ca_5_(PO_4_)_3_((OH)_0.2_F_0.8_)	25.20	0.93995	0.93995	0.68971
Ka60	Ca_5_(PO_4_)_3_((OH)_0.2_F_0.8_)	25.91	0.94044	0.94044	0.69088
Ka120	Ca_5_(PO_4_)_3_((OH)_0.2_F_0.8_)	28.58	0.94140	0.94140	0.69074
Ka240	Ca_5_(PO_4_)_3_((OH)_0.2_F_0.8_)	19.93	0.94088	0.94088	0.69110
Reference	CaCO_3_-Calcite (code amcsd 0000098)	-	0.49900	0.49900	1.79172
Ka0	CaCO_3_	2.34	0.49874	0.49874	1.70370
Ka30	CaCO_3_	1.95	0.49570	0.49570	1.71000
Ka60	CaCO_3_	2.45	0.49520	0.49520	1.71000
Ka120	-	-	-	-	-
Ka240	-	-	-	-	-
Reference	CaMg(CO_3_)_2_-Dolomite (code amcsd 0000086)	-	0.48150	0.48150	1.61190
Ka0	CaMg(CO_3_)_2_	7.03	0.48113	0.48113	1.6044
Ka30	CaMg(CO_3_)_2_	5.25	0.48138	0.48138	1.6039
Ka60	CaMg(CO_3_)_2_	4.64	0.48110	0.48110	1.6051
Ka120	CaMg(CO_3_)_2_	4.58	0.48120	0.48120	1.5984
Ka240	CaMg(CO_3_)_2_	5.83	0.48149	0.48149	1.6032
Reference	SiO_2_-Quartz (code amcsd 0006362)	-	0.49137	0.49137	0.54047
Ka0	SiO_2_	14.09	0.49146	0.49146	0.54057
Ka30	SiO_2_	15.48	0.49146	0.49146	0.54063
Ka60	SiO_2_	15.28	0.49146	0.49146	0.54063
Ka120	SiO_2_	15.30	0.49149	0.49149	0.54060
Ka240	SiO_2_	15.32	0.49146	0.49146	0.54058
Reference	CaHPO_4_, Monetite	-	0.6910	0.6627	0.6998
Ka0	-	-	-	-	-
Ka30	-	-	-	-	-
Ka60	CaHPO_4_	3.24	0.69500	0.65860	0.6980
Ka120	CaHPO_4_	8.02	0.69510	0.65860	0.6965
Ka240	CaHPO_4_	4.50	0.69510	0.66250	0.6970
Reference	β-Ca_2_P_2_O_7_ (cod2001132)	-	0.66858	0.66858	2.41470
Ka0	-	-	-	-	-
Ka30	-	-	-	-	-
Ka60	-	-	-	-	-
Ka120	β-Ca_2_P_2_O_7_	4.11	0.6702	0.6702	2.4239
Ka240	β-Ca_2_P_2_O_7_	3.36	0.6752	0.6752	2.4262

The data presented in Table 4 and Table 5 further clarify the results of the X-ray diffraction analysis. In addition to the phases listed in Table 3, new phases with similar diffraction lines have been identified. The diffractograms reveal the presence of carbonate-substituted fluorapatite with formula Ca_5_P_2.883_O_12.381_C_0.066_F_0.864_, which, after structure refinement, corresponds to Ca_9.7560_P_5.9520_O_23.9760_F_1.8840_ in the initial To0 and Ca_10_P_5.7660_O_24.7620_C_0.1320_F_1.7280_ in the triboactivated samples. The refinement of the structural formulae of the phases proves isomorphism in the apatite anion sublattice and substitution of a carbonate ion (by insertion of CO_2_ from air) at the positions of the phosphate ion as the amount of carbon atom increases in the apatite structure, and that of the phosphorus decreases with increasing activation duration. Apart from this isomorphic substitution, another isomorphic process is evidenced in the direction of the hexagonal axis along which the fluorine atoms are located. This is supported by the identification of hydroxyl fluorapatite with the formula Ca_5_(PO_4_)_3_((OH)_0.2_F_0.8_), which contains both fluorine and hydroxyl ions. Hydroxyl ions are most likely adsorbed from the environment by the fresh reaction surface that is revealed upon dry activation and reduction in crystallite size. Their presence implies alternation with the fluorine atoms along the hexagonal axis in a non-stoichiometric sequence [37], with the degree of substitution most likely increasing in favour of the hydroxyl ion with increasing triboactivation duration.

Solid-phase synthesis of ortho- and pyrophosphates was also established by Rietveld refinement in both sample series. Representatives of these groups of phosphates in the present study are CaHPO_4_-monetite and β-Ca_2_P_2_O_7_, the formation of which is found after a stronger tribological impact lasting more than 60 min. It is likely that the combination of the low degree of particle crystallinity and the relatively low content of these phases in the sample compositions makes it impossible to identify them without further processing of the results.

Both series of samples are characterized by a relatively high quartz content in the composition—over 10%. Quartz is a mineral of high hardness on the Mohs scale-7 [38], and this suggests that it will not undergo significant structural changes under the influence of tribochemical activation. The results obtained from both PXRD analysis and Rietveld refinement prove its stability. Quartz does not participate in solid-phase reactions, and its crystal-chemical parameters are kept relatively constant. Only a decrease in the intensity of the diffraction lines and their broadening at the base of the peaks is recorded.

Changes in the crystal lattice parameters can be characterized as significant, more so for the Toolse samples. The changes in lattice parameters are not uniform along the “a/b” and “c” axes. For isomorphically substituted phosphates, the general trend is a nonlinear decrease in the dimensions along the “a” and “c” axes for Ca_10_P_5.7660_O_24.7620_C_0.1320_F_1.7280_ in the triboactivated samples, as well as an expansion of the unit cell along the “a” and “c” axes for Ca_5_(PO_4_)_3_((OH)_0.2_F_0.8_). Most significantly, this change is reported for the lattice parameters of To60.

A characteristic feature of the carbonate-bearing phases, calcite and dolomite, is an overall shrinkage of the unit cell by approximately 5% along the “a” “c” (To60) axes compared to the reference specimen (Table 4 and Table 5).

The described trends in lattice parameter changes for the Kabala samples are similar but less pronounced, with smaller deviations from the values compared to the reference specimen parameters shown in Table 5.

For the newly formed monetite and calcium pyrophosphate phases, the general trend for parameter changes is contraction along the “a” axis and expansion along the “c” axis. Another feature is the steady increase in the content of these phases in the HEM-activated sample compositions at the expense of a decrease in the fluorapatite content of the initial compositions. Thus, in spite of the constancy of the phase composition identified in Figure 6a,b, its dynamic variation, both in terms of isomorphically substituted phases and their contents, and in terms of changes of their lattice parameters under the influence of the tribochemical activation, is detected by Rietveld refinement. Two trends are evident, the first: better pronounced structural changes through the numerical values obtained from Rietveld refinement for the Toolse samples and the second: the nonlinear variation of the crystal lattice parameters for the triboactivated samples. All the numerical values of the parameters for the samples at 240 min of triboactivation (To240 and Ka240) approach either the parameters of the initial samples or those of the samples with short activation times (To0, To30, Ka0 and Ka30).

### 4.5. FTIR Spectroscopy

**Figure 8:**
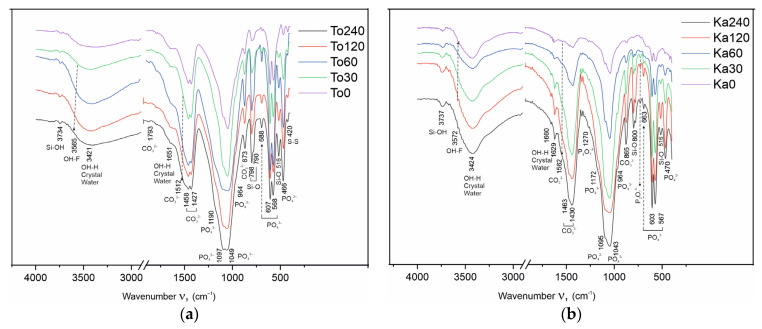
(**a**) FTIR spectra of non-activated and triboactivated Toolse samples (30–240 min); (**b**) FTIR spectra of non-activated and triboactivated Kabala samples (30–240 min).

The FTIR analysis results are given in Figure 8a,b and Table 6. The changes resulting from the tribochemical treatment were also studied by FTIR spectroscopy. In the obtained spectra of the Toolse and Kabala samples, the vibrational peaks of the phosphate (PO_4_^3−^), carbonate (CO_3_^2−^), hydroxyl (OH^−^), and fluoride (F^−^) groups were observed. In Figure 8, the absorption bands that belong to the spectra of the functional group that enter into the composition of the compounds are marked with different colors. Table 6 presents the vibrational modes that characterize the full FTIR spectra of the chemical bonds in the individual functional groups and the changes in them under the influence of the triboactivation.

The phosphate group (PO_4_^3−^) in the spectra of all Toolse and Kabala samples reveals two strong absorption bands in the range of asymmetric stretching (ν_3_) peaking at 1094 and 1043 cm^−1^. A peak at 964 cm^−1^ originates from symmetric ν_1_(PO_4_^3−^) stretching, while the peak at 604 cm^−1^ from the ν_4_(PO_4_^3−^) bending in apatite [39].

In addition to the absorption bands of the phosphate ion, strong vibrational bands of the carbonate group are detected in the spectra (Figure 8a,b). The characteristic vibrations of the carbonate ion determine its positions in the different carbonate-containing compounds. Calcite (CaCO_3_) reveals strong absorption peaks at 1430 and 870 cm^−1^ due to antisymmetric ν_3_(CO_3_^2−^) stretching and out-of-plane ν_2_ vibrations of CO_3_^2−^, as well as weaker signals due to overtones and combination bands (ν_2_ + ν_4_) around 2520 cm^−1^. Almost in the same positions fall the dolomite bands, as the offset of ν_4_(CO_3_^2−^) in the range 728–729 cm^−1^ corresponds to the absorption streak of the carbonate ion in the composition of dolomite. In the spectra of the activated samples in the interval 1510–1560 cm^−1^, a slight broadening as a shoulder is found in the left half of the carbonate ion band, corresponding to an asymmetric ν_3_(CO_3_^2−^) stretching vibrational mode. The occurrence of this peak is explained by the tribochemical effect, which is the cause of revealing a fresh reaction surface on which CO_2_ is adsorbed from the air. The absorbed CO_2_ creates new bonds in the apatite structure and this explains the appearance of new bands or broadening of already existing bands in the spectra [40].

The observed peaks near 799 and 780 cm^−1^ are characteristic of the presence of quartz (SiO_2_), along with the peaks at 695, 515 cm^−1^ [41].

The asymmetric band of sulfide sulfur ν(S-S) in the 420–425 cm^−1^ region is confirmed by previous studies [25] of FeS_2_ in the composition of the Toolse samples.

The broad asymmetric band in the 3200–3700 cm^−1^ interval reveals the presence of water molecules in the composition of the samples from both deposits. The positions of the vibrational bands of structurally bound and crystallized water appear in the FTIR spectra at different wavenumber values. Their positions provide additional information about the distribution of water in the samples and the type of binding in the structure. No phases are present in the composition of the samples in which water participates as crystallization water. The broad band with maximum at 3200–3350 cm^−1^ is ascribed to stretching vibrations ν(OH) and 1640–1650 cm^−1^ bending vibrations ν(HOH) of water. These water molecules are most likely physically bound by weak intermolecular bonds. Similar is the Si-OH bond in the asymmetric ν(OH^−^) band in the 3737–3739 cm^−1^ range. This band characterizes the Me-OH bond, where Me is a cation (in the present case, Me = Si) with the position of the band depending on the valence and radius size of the cation. The larger the radius and valence of the cation, the position of the asymmetric valence vibration ν(OH) is shifted towards the larger wavenumbers in the spectra [42].

## 5. Discussion

The studies carried out in the present work evaluate the impact of tribochemical activation as an alternative method for enhancing the chemical reactivity of phosphorites from the Toolse and Kabala deposits in Estonia, providing an opportunity to analyze the results obtained.

The effect of deliberately created structural destabilization of the solid is evaluated by changes in the structure by chemical analysis and microstructural methods. The results obtained from the applied impact with a duration of 10 to 240 min of grinding with 20 mm in size grinding bodies of alloy Cr-Ni steel give grounds to summarize the results obtained in three groups:

### 5.1. First Group-Isomorphic Transformations

The results obtained from the conducted investigations demonstrate the occurrence of isomorphic substitutions in the anionic sublattice in the apatite structure. Anionic isomorphism is associated with two types of substitutions—in the positions of the phosphate ion and in the positions of the fluorine atom along the hexagonal axis. In the present paper, the XRF analysis results of the HEM-activated To30–To240 and Ka30–Ca240 samples provide initial information on possible isomorphic trends in the contents of the major components, reporting a 44.63% decrease in the fluorine content for To240. This is confirmed by the linearization of the results performed (Figure 3a,b, Table 1 and Table 2), confirming the non-symbolic nature of the decrease in F atom content with increasing HEM duration. In this case, when F is redistributed to other phases, its general content should be preserved. The recorded decrease is an indication of it leaving the system, its place being taken by another anion, namely the hydroxyl. The source of the hydroxyl ion is the air environment in which the experiments are carried out. In confirmation of the XRF analysis results are the XRD measurements (Figure 6a,b, Table 4 and Table 5). Rietveld refinement data establish isomorphically substituted fluor-hydroxyl apatite with the general formula Ca_5_(PO_4_)_3_((OH)_0.2_F_0.8_), in which OH and F share a single crystallographic position along the hexagonal axis. The results are confirmed in previous studies for triboactivated [20] and non-activated apatites [43]. The third confirmation is obtained in the spectra of the HEM samples from the FTIR measurements (Figure 8a,b, Table 6). In the range of O-H stretching vibrations, the strong and broad peak centered at 3435 cm^−1^ confirms the presence of water molecules. These peak masks the appearance of other vibrations due to the hydroxyl group in fluor-hydroxyapatite, which are expected to be present at about 3535 cm^−1^ because the interaction of fluorine anion with hydrogen leads to a shift of the OH-stretching vibration from 3572 cm^–1^ to 3535 cm^–1^ in the F-OH apatite solid solution [44,45]. Another evidence for the presence of hydroxyl apatite and hydroxyl group should be the OH-libration in hydroxylapatite at 632 cm^−1^. Such a peak is not observed in the spectrum. It is known that increased fluorine content in hydroxylapatite causes a shift to higher frequencies [37,46]. At higher frequencies, a peak characteristic of fluor-hydroxyapatite is registered around 670–660 cm^−1^.

A manifestation of the second type of isomorphism in apatite is the incorporation of a carbonate group at the positions of the phosphate ion or in the channels along the hexagonal axis. Sources of carbonate ions can be impure carbonate-bearing minerals in apatite formation (e.g., for phosphorites, this is the marine environment) [47] or CO_2_ from the air in the case of HEM activation [40]. The results obtained from the microstructural analysis prove the presence and formation of two types of isomorphous substitutions in the apatite structure with carbonate ions. The first type, known as B-type carbonate-apatite is formed as a result of the genesis of the ore in nature and represents the incorporation of carbonate ions into the positions of the phosphate group. Evidence for its presence in the Toolse and Kabala samples is provided by Rietveld refinement results (Table 4 and Table 5) and FTIR spectroscopy (Figure 8a,b, Table 6). Rietveld refinement data establish isomorphically substituted carbonate-fluorine apatite with the general formula Ca_9.7560_P_5.9520_O_23.9760_F_1.8840_, already in initial non-activated samples (To0 and Ka0), as after HEM activation, the amount of carbonate ions increases, and the formula is transformed into Ca_10_P_5.7660_O_24.7620_C_0.1320_F_1.7280_.

In FTIR, B-type carbonate apatite is characterized by strong peaks at 1429 cm^−1^, as well as a shoulder on the higher frequency near 1470 cm^−1^ and 1540 cm^−1^. The carbonate ion CO_3_^2−^ can substitute for OH^−^ or F^−^ in the channels (A-type) in the crystal structure of flourapatite. Carbonate-apatite reveals well-resolved peaks due to asymmetric ν_3_(CO_3_) stretching and out-of-plane ν_2_ vibrations of CO_3_ impurities in apatite, depending on the type of isomorphic substitution (A-type; B-type; AB-type) in the range 1425–1550 cm^−1^ and 870 cm^−1^, respectively [48].

The main result of the studies of isomorphic transformations in the structure of the Toolse and Kabala samples is that they are representatives of B-type carbonate apatites. After HEM activation for up to 240 min, A-type hydroxyl carbonate apatite with altered lattice parameters—nonlinear contraction along the “a” axis and expansion along the “c” axis—is formed.

### 5.2. Second Group-Solid-Phase Synthesis

The resulting isomorphic transformations and changes in the lattice parameters of the unit cell led to the accumulation of defects and stresses in the structure of apatite and impurity minerals in the composition of samples from both deposits.

The essence of the HEM activation method is related to the application of shock-intensive energy impact on a solid body in a mechanical friction process in a planetary mill-type triboreactor. As a result of the selection of appropriate parameters of activation, type and size of grinding bodies, degree of filling of reaction vessels, duration of applied impact, intensity of rotation, and conditions for accumulation of mechanochemical energy in the solid body are created. The accumulated energy destabilizes the structure of the solid through the accumulation of induced structural defects, amorphization, and metastable polymorphic forms, and leads to transformation into an energy-rich metastable state. Such a solid state is unstable and, upon further impact, such as heating or chemical attack, spontaneously relaxes into an energetically more stable state [49,50]. It is in the relaxation of energy that it is possible for this energy to be used to support chemical reactions, solid-phase synthesis, etc. [51]. The Rietveld refinement microstructural analysis results (Table 4 and Table 5) and the FTIR spectra (Figure 8a,b, Table 6) also confirm the occurrence of solid-phase synthesis reactions. Calcium ortho- and pyrophosphates were identified, confirming the catenation rule that P-O-P chains from hydrogen ortho- or hydrogen phosphates proceed first through the formation of ortho-phosphates. In the FTIR spectra, the pyrophosphate group is identified by low intensity bands at 727–729 cm^−1^ of the symmetric vibrational ν_1_(O-P-O) vibration and 1269–1270 cm^−1^ of the asymmetric ν_3_(P-O-P) vibrational ν_3_(P-O-P) vibration in P_2_O_7_^4−^. In our previous studies, the orthophosphate formation stage has not been proven. In most of the previous studies, the formation of pyrophosphates has been established directly. Probably, the presence of higher concentrations of quartz in the compositions slows down the tribochemical effect, except that it becomes possible to establish the formation stage of CaHPO_4_.

### 5.3. Third Group-Chemical Activity

The results obtained from the chemical analysis and mathematical processing unequivocally proved an increase in the solubility of P_2_O_5_ in 2% citric acid solution. Moreover, the increase was found already at low activation duration—about 30 min (Figure 4). This is an important parameter for determining the optimum treatment conditions for materials. The rapid rate of solubility increase is evidence that initially, the effect of particle size reduction and the revelation of a fresh reaction surface have a leading effect on solubility and chemical reactivity. The conclusion is supported by the specific surface area measurement results (Figure 5), which, to some extent, replicate the rapid rate of increase in SSA during the low activation periods, about 30–60 min, followed by a rapid rate of decrease in SSA. Chemical analysis demonstrates the retention of high P_2_O_5_ solubility values up to 240 min of HEM, but the mathematical model of the exponential equation predicts that with longer activation, solubility will begin to decrease, or repeat the SSA measurement results, but at a slower rate. This implies that at high HEM times, the influence of structural defects and stresses is dominant, leading to solid-phase fusion reactions and the formation of new phases. A decrease in solubility and a decrease in specific surface area result from particle agglomeration. This leads to a limitation of the fresh reaction surface and to a decrease in the numerical values of the studied characteristics. These secondary effects of HEM activation are important for specifying the activation mode and selecting its optimum conditions.

The studies carried out and the results obtained on the triboactivation of phosphorites from deposits in Estonia warrant an analysis of the effect of the applied method on the crystal-chemical properties of the samples studied and the potential for their application, which is the aim of the present work.

As a result of this structure destabilization, the samples from both sites were found to exhibit similar transformations as displays of anionic isomorphism along the hexagonal axis at the F^−^ atom positions with substitution by an OH^−^ ion, with the degree of OH^−^ substitution increasing with increasing activation time (Figure 8a,b, Table 1 and Table 2). This modification results in the formation of mixed hydroxyl-fluorapatite (Figure 7a,b). In the channels of the apatite structure near the hexagonal axis, CO_2_ incorporation from air is established, which is associated with the formation of carbonate-type A apatite with a gradual increase in carbon atom content (Table 4 and Table 5). The occurrences of isomorphism and structural deformation in the apatite structure also lead to changes in the lattice parameters of the elementary cell, mostly in Carbonate fluorapatite and Fluor hydroxyl apatite (Table 4 and Table 5). HEM activation also leads to the realization of solid-phase reactions with the formation of CaHPO_4_-monetite and also β-Ca_2_P_2_O_7_. The formation of monetite, according to the results obtained, starts at shorter activation periods and can be assumed to form before dicalcium diphosphate. Thus, the rule that pyrophosphate formation precedes orthophosphate formation is confirmed, the main conclusion being the disruption of the apatite structure and solid-phase synthesis of new phases upon activation.

Further evidence of the tribochemical effect achieved was an increase in the solubility of the activated samples from both series in 2% citric acid solution from 7.5–8.0% (To0 and Ka0) to 55.5–55.8% (To240 and Ka240) (Figure 4a,b). This is evidence of an increase in chemical reactivity, and this result proves the feasibility of using the HEM method to treat natural samples in order to use them directly for the needs of bio-agriculture as activators, improvers, or fertilizers. Contributing to the activation effect of the phosphorite samples are the impurity minerals—carbonates (calcite and dolomite) and quartz, which are present in greater amounts than the other impurities such as pyrite, K-feldspar, muscovite, sanidine, etc. (Table 1, Table 2, Table 4 and Table 5, Figure 6) [5]. Through the chemical composition and nature of these minerals (hardness, chemical reactivity, etc.) they can exhibit an accelerating or retarding effect in triboactivation.

The results of the crystal-chemical studies also demonstrate some differences in the behavior of the Toolse and Kabala deposits. For example, the tribological effect is more pronounced for the major mineral apatite in samples from the Toolse deposit compared to those from Kabala. The type and relative number of isomorphous phases are analogous, but the products of the solid-phase reactions (monite and pyrophosphate) in the Toolse deposit samples are present in greater amounts for the same duration of activation (Table 4 and Table 5). Calcite and dolomite, which are soft minerals (3.5 and 3.0 on the Mohs scale), are gradually transformed into X-ray amorphous minerals due to a decrease in their crystallinity and crystallite size. They are carbonate-bearing minerals, and no negative effect on isomorphous transformations and solid-phase reactions is found from the results of the studies. It is expected that the effect of HEM activation would be reflected in the direct use of activated samples in soils where carbonate minerals would play a role in regulating pH and acidity. A reliable quantification of the carbonate ion content of the apatite structure cannot be presented at this stage. With continued research and the use of quantitative methods such as thermal methods, it will be possible to estimate the carbonate ion content as well as the thermal stability of the various isomorphically substituted phases.

The results presented here on samples from two phosphorite deposits in Estonia are part of an extensive study by the team to evaluate the impact of the HEM activation method on the crystal-chemical and thermal properties of phosphorites from different origins—Europe (Estonia), North Africa (Tunisia), West Asia (Syria) [20,52,53]. The results of these studies present a confirmation of the results of our current studies with respect to the occurrences of isomorphism and enhancement of the phosphorites’ chemical reactivity. Differences in the achieved activation effect are explained by differences in the content of impurity minerals, especially quartz, and the conditions of HEM activation—duration, type and size of grinding bodies, etc.

### 5.4. Ecological Applications

As a result of the studies carried out to evaluate the effect of tribochemical activation on samples from two phosphorite deposits in Estonia, some conclusions and recommendations can be drawn regarding the potential environmental applications of the chosen raw material treatment approach.

The obtained results prove that the use of the dry milling method achieves an increase in the chemical reactivity of the phosphorite ore. This makes it possible to eliminate the acid treatment stage with its associated severe environmental problems [54]. At the same time, solutions can be sought for direct application in soils under certain technical possibilities, which can have a number of advantages, such as:

− Elimination of the acid treatment stage with environmental problems, costly mineral raw materials, and energy costs. Costs of preparing raw materials for direct use remain;
− Application for bio-agricultural purposes due to the natural origin of phosphorite ore;
− Ability to use lower quality raw materials (lower P_2_O_5_ content) with reduced fuel costs;
− Variety of application purposes depending on the composition, impurities, and processing—as a soil improver, natural mineral fertilizer, activator;
− Increases nutrient uptake and improves soil structure, thereby improving soil fertility and controlling soil degradation, particularly nutrient leaching (depletion).

The advantages listed must also be reconciled with the treatment conditions, duration of activation, avoidance of compositional variability, and avoidance of contamination with heavy elements and radionuclides.

## 6. Conclusions

This work presents the results of HEM tribochemical activation of phosphorite samples from two deposits, Toolse and Kabala, from Estonia, for times ranging from 10 min to 240 min with 20 mm Cr-Ni grinding bodies (made of alloy steel).

The aim of the study is to determine the choice of method and appropriate conditions for the treatment of phosphorite ore under which the reactivity of the main mineral apatite and mineral additives on the carbonate–quartz–apatite system for ecological applications is increased—acid-free preparation of improvers and fertilizers, raw material for REE extraction, soil improvers, activators, etc.

The enhancement of chemical reactivity is evaluated by a complex of analytical methods that include chemical analysis, XRF and PXRD, BET, and FTIR. With the results obtained, three groups of induced changes in the apatite structure were observed—anionic isomorphism with formation of carbonate–hydroxyl–fluorine apatite, solid-phase synthesis—formation of calcium-hydrogen ortho-phosphate and dicalcium pyro-phosphate. The chemical reactivity is expressed in the increase of P_2_O_5_ solubility in 2% citric acid solution to 54–56% P_2_O_5_^a^/P_2_O_5_^tot^ as a result of the accumulated induced defects and stresses in the apatite crystal structure and the accumulated energy in the solid. Based on the result of the increase in reactivity of the samples, it is possible to apply them directly as improvers, activators, or fertilizers for the purposes and needs of agro-biogardening.

## Figures and Tables

**Table 1:** XRF analysis data for the non-activated (sample To0) and activated (To10–To240) Toolse samples.

Content of Components	Sample To0	Det. Limit	Sample To10	Det. Limit	Sample To30	Det. Limit	Sample To60	Det. Limit	Sample To120	Det. Limit	Sample To240	Det. Limit
	(mass%)											
F	3.54	0.66066	2.71	0.63850	2.20	0.60897	2.23	0.58454	2.11	0.58505	1.96	0.55566
CaO	49.70	0.02372	49.34	0.01626	49.09	0.01675	49.57	0.01654	48.75	0.01661	49.19	0.01645
P_2_O_5_^tot^	26.77	0.03063	26.36	0.02080	26.34	0.02101	26.31	0.02012	26.12	0.02069	25.33	0.02068
SiO_2_	16.08	0.03933	14.44	0.02525	14.32	0.02523	15.55	0.02559	14.58	0.02471	15.77	0.02659
Na_2_O	0.47	0.07795	0.42	0.05085	0.42	0.04845	0.49	0.04785	0.54	0.04997	0.45	0.05354
K_2_O	0.44	0.01467	0.43	0.00939	0.41	0.00985	0.40	0.00936	0.41	0.00976	0.38	0.00963
Al_2_O_3_	1.09	0.01366	0.89	0.00887	0.86	0.00850	0.77	0.00847	0.81	0.00936	0.76	0.00888
Fe_2_O_3_	2.23	0.01698	2.70	0.00974	2.66	0.00644	2.78	0.01058	2.67	0.00963	2.88	0.01058
SO_3_	2.18	0.01126	2.27	0.00696	2.35	0.00659	2.42	0.00700	2.40	0.00761	2.43	0.00717
MgO	0.82	0.04729	0.68	0.03177	0.78	0.03072	0.66	0.03059	0.65	0.03259	0.68	0.03042

**Table 2:** XRF analysis data for the non-activated (sample Ka0) and activated (Ka10–Ka240) Kabala samples.

Content of Components	Sample Ka0	Det. Limit	Sample Ka010	Det. Limit	Sample Ka030	Det. Limit	Sample Ka060	Det. Limit	Sample Ka120	Det. Limit	Sample Ka240	Det. Limit
	(mass%)											
F	2.94	0.72344	2.90	0.68286	2.65	0.67764	2.63	0.41906	2.89	0.61774	1.90	0.56198
CaO	53.60	0.01686	55.72	0.01720	54.11	0.01676	54.17	0.01674	55.03	0.01691	53.77	0.01669
P_2_O_5_^tot^	27.50	0.02081	29.24	0.02081	28.68	0.02107	28.11	0.02122	28.39	0.02112	27.84	0.02115
SiO_2_	9.76	0.02243	9.40	0.02192	9.78	0.02193	9.94	0.02192	10.82	0.02158	10.93	0.02170
Na_2_O	0.38	0.04842	0.43	0.05611	0.53	0.05294	0.39	0.05063	0.51	0.05248	0.41	0.05047
K_2_O	0.12	0.00936	0.14	0.00942	0.13	0.00936	0.13	0.00938	0.13	0.00933	0.13	0.00943
Al_2_O_3_	0.45	0.00903	0.26	0.00813	0.22	0.00824	0.18	0.00801	0.21	0.00781	0.20	0.00765
Fe_2_O_3_	2.52	0.00967	2.06	0.01122	1.93	0.00898	2.06	0.00835	2.08	0.01067	2.11	0.00810
SO_3_	0.44	0.00548	0.44	0.00580	0.45	0.00558	0.45	0.00653	0.50	0.00550	0.49	0.00668
MgO	1.63	0.03440	1.64	0.03161	1.41	0.03375	1.33	0.03225	1.40	0.03456	1.37	0.03332

**Table 3:** Identified phases in the samples according to PXRD analysis results.

Description	Sample	Identified Phases (Formula, ICDD Card Number)
Fluorapatite	Toolse 0–240, Kabala 0–240	Ca_5_F(PO_4_)_3_, #15–0876
Calcite		CaCO_3_, #47–1743
Dolomite		CaMg(CO_3_)_2_, #36–0426
Quartz		SiO_2_, #46–1045
Muscovite	Toolse 0–30	Al_11.68_Fe_0.32_K_2.40_Si_12_O_48_, #34–0175
Gypsum	Toolse 0	CaSO_4_·2H_2_O, #33–0311

**Table 6:** FTIR spectroscopy results—band positions and assignments.

Samples	ν_1_ (Symmetric Stretching) *, (cm^−1^)	ν_2_ (Symmetric Bending) *, (cm^−1^)	ν_3_ (Asymmetric Stretching) *, (cm^−1^)	ν_4_ (Asymmetric Bending) *, (cm^−1^)	Bond	Description
1. Minerals in phosphorite ore						
To0-To240 Ka0-Ka240	964–966	468–471	1040–1050 1097–1098 1175–1109	605–607 570–574	P-O in PO_4_^3−^	Fluorapatite
	-	-	1458–1460	-	C-O in CO_3_^2−^	CO_3_^2−^ in fluorapatite type B
	3374 3416–3433	1640–1650	-	-	OH-H	Crystal water
	-	-	3527–3574	-	OH-F	Structurally bound water in fluorapatite
To0-To240	-	870–873	1429–1460	712	C-O in CO_3_^2−^	Calcite
	-	1793–1796	1870		C-O in CO_3_^2−^	(2ν_2_) CO_3_^2−^ (2ν_3_) CO_3_^2−^ in Calcite
Ka30-Ka240	-	875–878	1429–1442	728–729	C-O in CO_3_^2−^	Dolomite
To0-To240 Ka0-Ka240	690–696 779–781 798–799	468–471	1184–1190	513–517	Si-O	Quartz
To0-To240	-	-	420–425	-	S-S	Pyrite
To30-To240 Ka0-Ka240	-	-	3737–3739	-	Si-OH	Free sylanol
2. Isomorphic substitution in fluorapatite						
To30-To240 Ka30-Ka240	964–966	468–471	1040–1050 1097–1098 1175–1109	605–607 570–574	P-O in PO_4_^3−^	Fluorapatite with CO_3_ incorporation on the hexagonal axis in fluorapatite type A
	-	-	1510–1529 1556–1560	-	C-O in CO_3_^2−^	
	-	-	3544–3574 3575–3577	-	OH-F	Substitution of F with OH-Hydroxyl-fluorapatite
3. Solid-state synthesis phase						
Ka30-Ka240	727–729	-	-	-	O-P-O in P_2_O_7_^4−^	β-Ca_2_P_2_O_7_
	-	-	1269–1270	-	P-O-P in P_2_O_7_^4−^	

* In the case of CO_3_^2−^, PO_4_^3−^ and SiO_4_^4−^ groups, they belong, respectively to ν_1_, ν_2_, ν_3_, ν_4_ types of vibrational modes.

## Data Availability

The original contributions presented in this study are included in the article/Supplementary Material. Further inquiries can be directed to the corresponding author.

## Supplementary Materials

The following supporting information can be downloaded at: https://www.mdpi.com/article/10.3390/ma18153508/s1.
