# Supplementary material for: Impact of High Energy Milling and Mineral Additives on a Carbonate–Quartz–Apatite System for Ecological Applications
Source: Materials (Basel). 2025 Jul 26;18(15):3508. doi: 10.3390/ma18153508 (PMC12347609; doi:10.3390/ma18153508)
Supplement: Supplementary file 1 [file materials-18-03508-s001.zip › materials-3739312-supplementary.pdf]

Impact of High Energy Milling and Mineral Additives on a Carbonate-Quartz-Apatite System for Ecological Applications

V. Petkova [1], K. Mihaylova [1], E. Serafimova [2], R. Titorenkova [1], L. Tsvetanova [1] and A. Trikkel [3]
[1]Institute of Mineralogy and Crystallography "Acad. Ivan Kostov", Bulgarian Academy of Sciences, Acad. G. Bonchev Str., bl.107, 1113 Sofia, Bulgaria;
[2]University of Chemical Technology and Metallurgy, 1756, 8 Kl. Ohridski Blvd., Sofia, Bulgaria,
[3]Tallinn University of Technology, Ehitajate tee 5, 19086, Tallinn, Estonia;
*Correspondence: vpetkova@clmc.bas.bg

Rietveld refinement of non-activated and HEM activated samples of phosphorite of Toolse deposite, Estonia

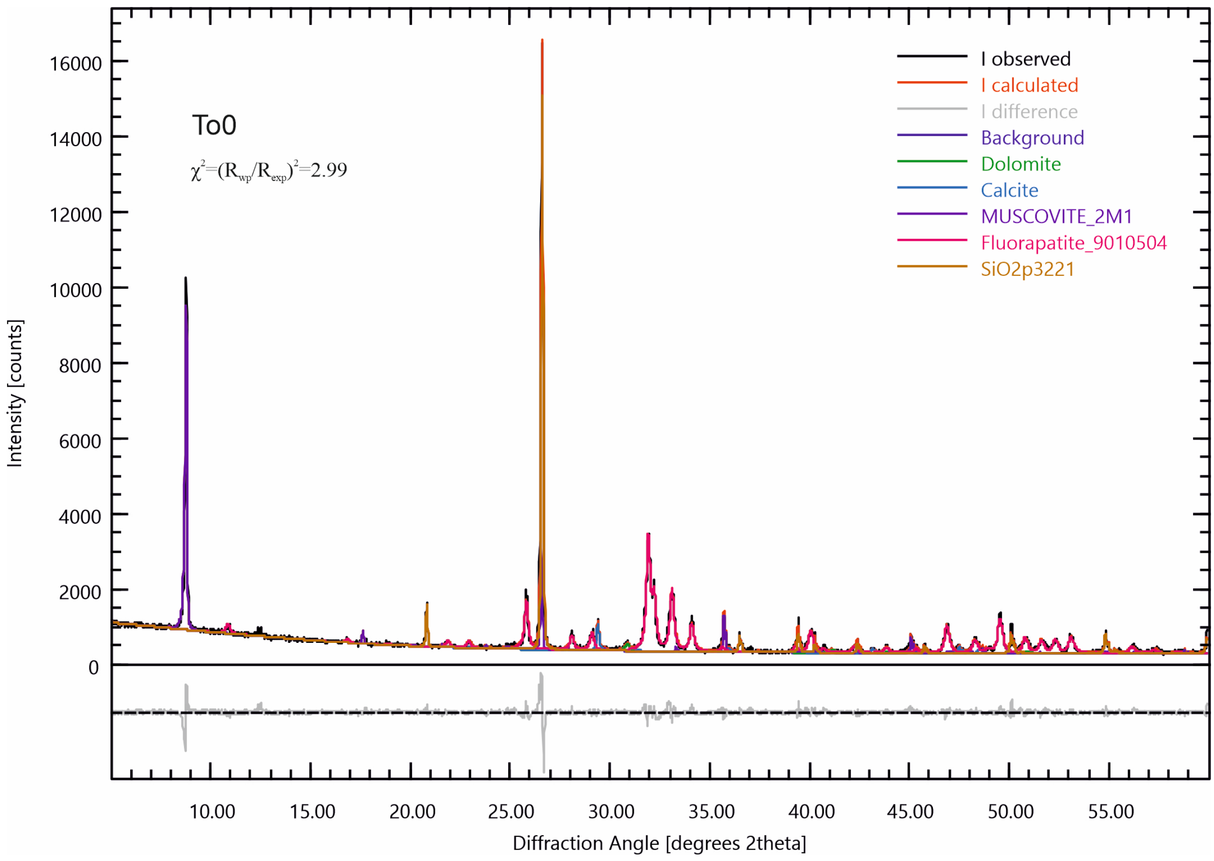

PXRD pattern refinement of non-activated Toolse sample

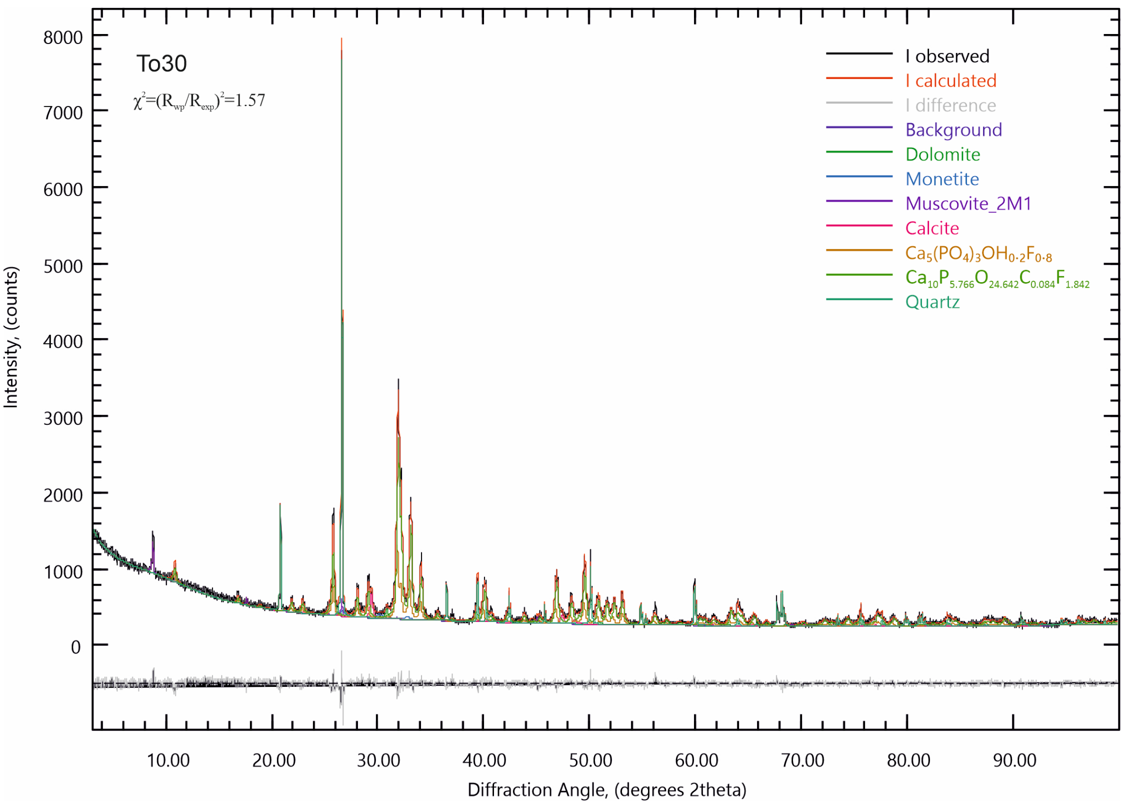

PXRD pattern refinement of triboactivated Toolse sample for 30 min HEM activation

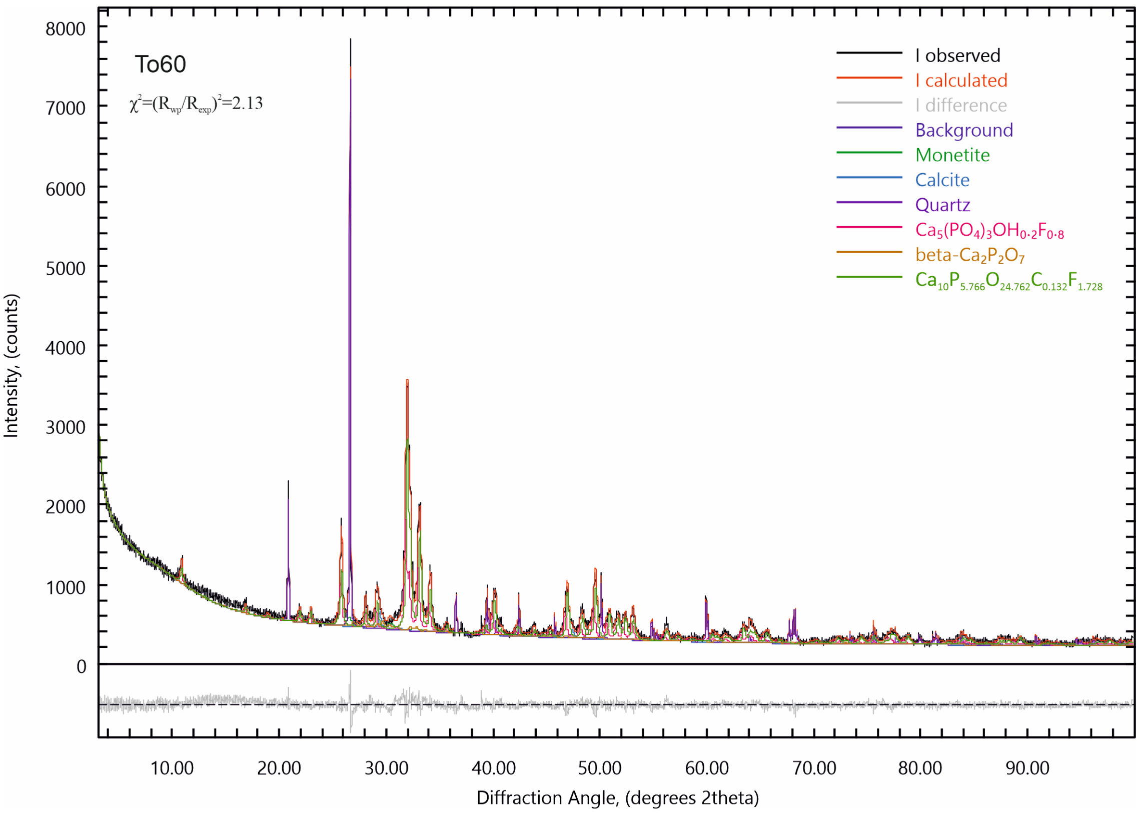

PXRD pattern refinement of triboactivated Toolse sample for 60 min HEM activation

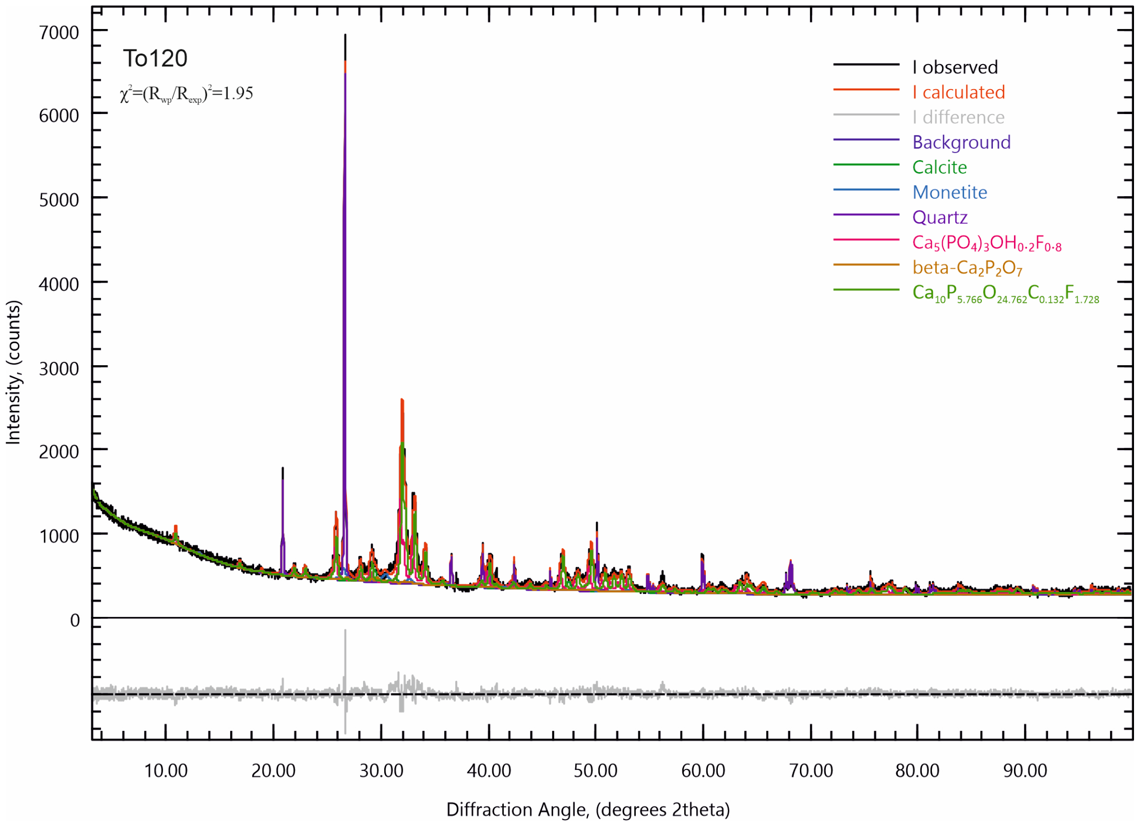

PXRD pattern refinement of triboactivated Toolse sample for 120 min HEM activation

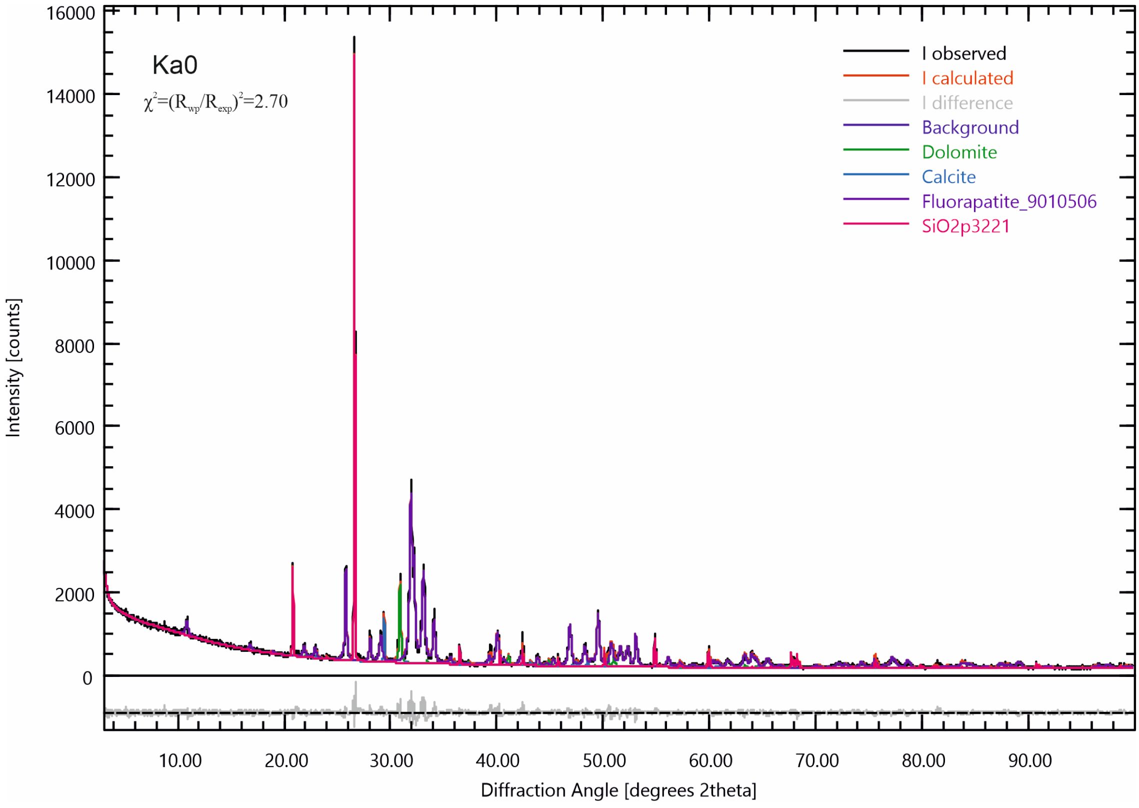

PXRD pattern refinement of non-activated Kabala sample

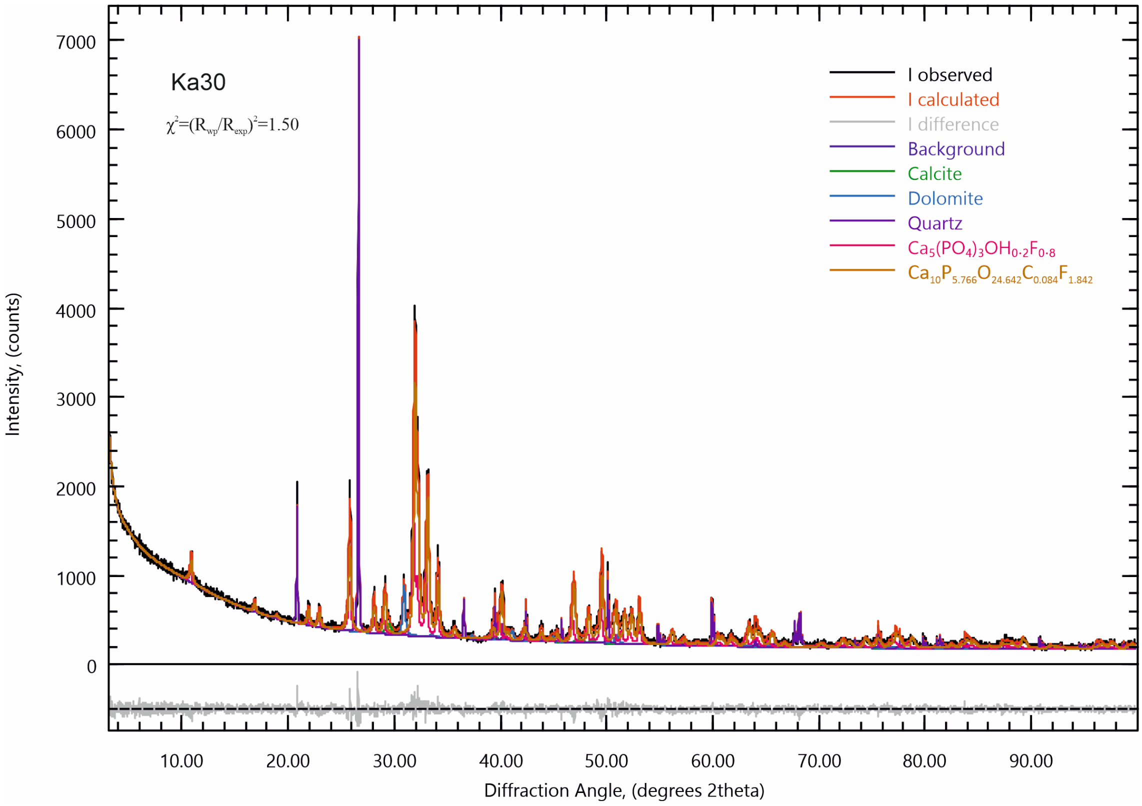

PXRD pattern refinement of triboactivated Kabala sample for 30 min HEM activation

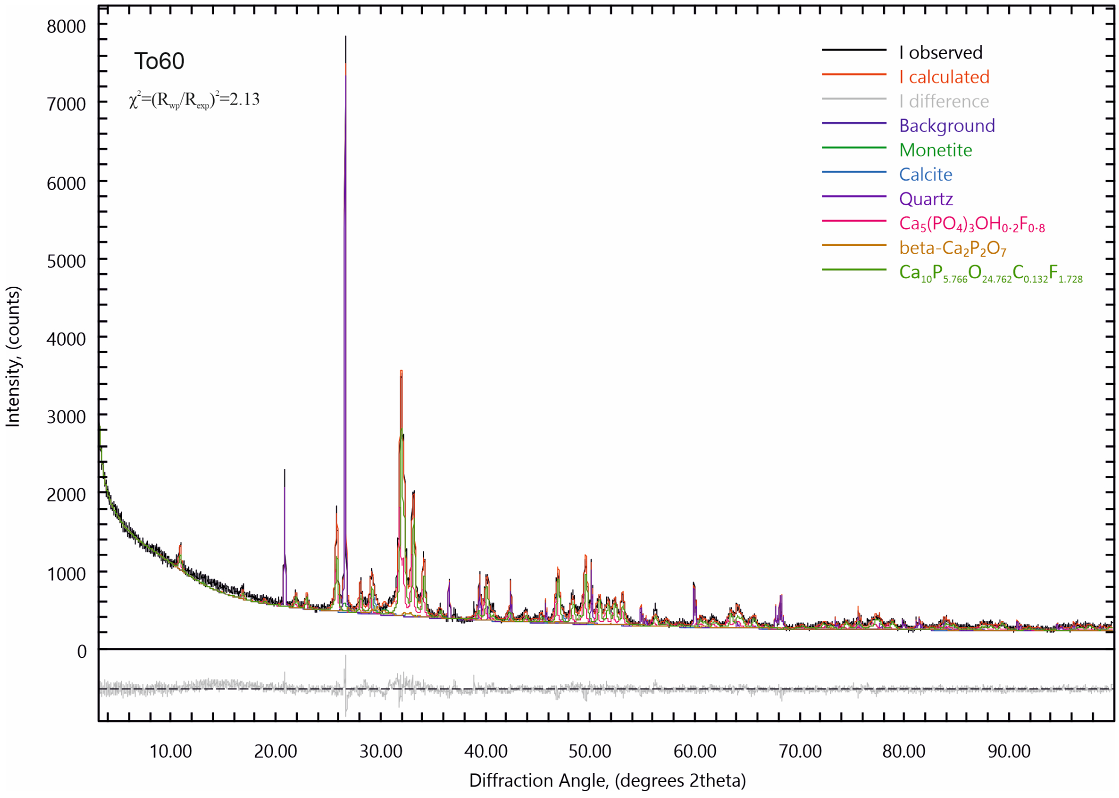

PXRD pattern refinement of triboactivated Kabala sample for 60 min HEM activation

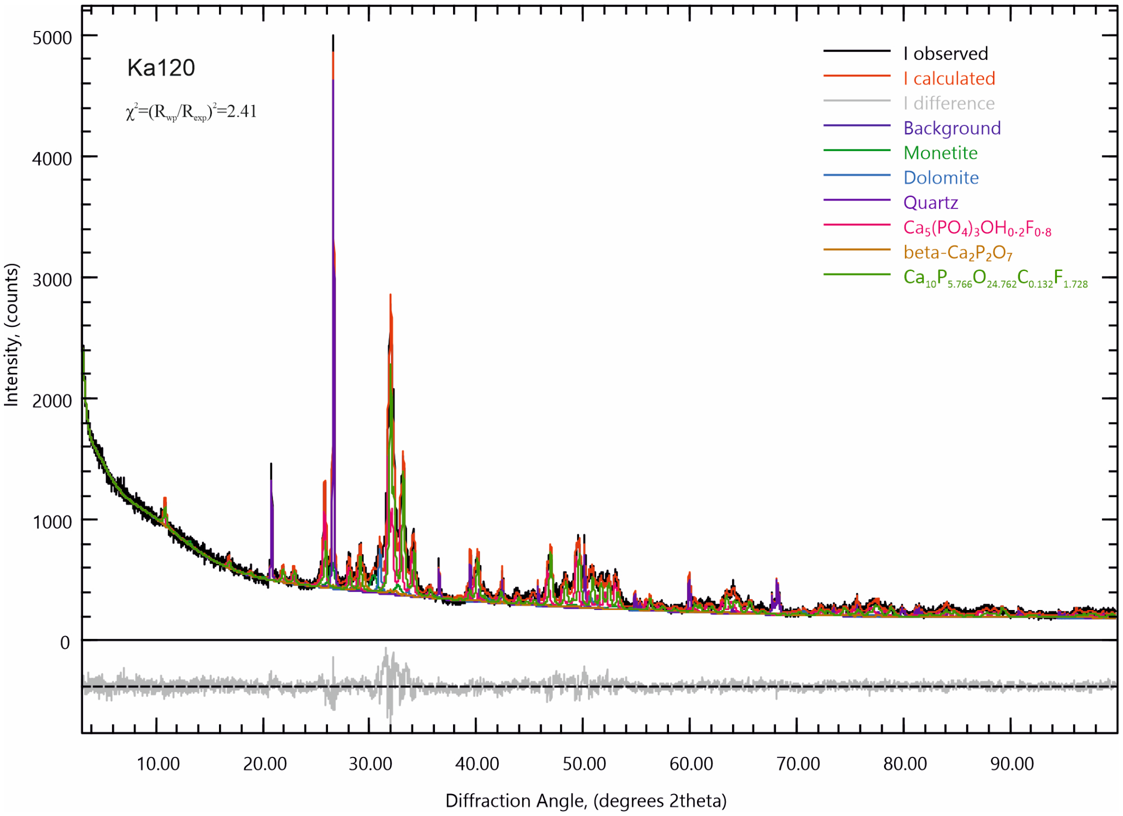

PXRD pattern refinement of triboactivated Kabala sample for 120 min HEM activation

Abbreviations used:

**Abbreviation**	**Description**
HEM	High-energy milled
To0 raw	Phosphorite, inactivated from Toolse deposite, Estonia
Ka0 raw	Phosphorite, inactivated from Kabala deposite, Estonia
CFAp, B-type	Carbonate-fluorapatite B-type, characterizes carbonate ion substitution in the phosphate ion positions in the apatite structure
CFAp, A-type	Carbonate-fluorapatite A-type, characterizes carbonate ion substitution in the c-axis channel in the apatite structure
COHFAp	Hydroxyl-carbonate-fluorapatite $Ca_{10}(PO_4)_4(CO_3)_3OHF$
CAp	Carbonate-fluorapatite
PXRD	Powder X-ray diffraction
WD-XRF	Wavelength Dispersive X-Ray Fluorescence
FTIR	Fourier Transformed Infrared
To30/Ka30	Phosphorite, activated for 30 min with 20 mm Cr-Ni steel milling bodies from Toolse/Kabala deposite, Estonia
To60/Ka60	Phosphorite, activated for 60 min with 20 mm Cr-Ni steel milling bodies from Toolse/Kabala deposite, Estonia
To120/Ka120	Phosphorite, activated for 120 min with 20 mm Cr-Ni steel milling bodies from Toolse/Kabala deposite, Estonia
To240/Ka240	Phosphorite, activated for 240 min with 20 mm Cr-Ni steel milling bodies from Toolse/Kabala deposite, Estonia
$P_2O_5^{tot}$	Общо съдържание на фосфор, преизчислен като P_2O_5
$P_2O_5^{a}$	P_2O_5 determined by direct extraction by 2% citric acid
$CaH_2P_2O_7$	Calcium dihydrogen pyrophosphate or Calcium dihydrogen diphosphate
$Ca_2P_2O_7$	Dicalcium pyrophosphate or Dicalcium diphosphate
